# Cocreation with Dutch patients of decision‐relevant information to support shared decision‐making about adjuvant treatment in breast cancer care

**DOI:** 10.1111/hex.13510

**Published:** 2022-05-17

**Authors:** Inge S. van Strien‐Knippenberg, Marieke C. S. Boshuizen, Domino Determann, Jasmijn H. de Boer, Olga C. Damman

**Affiliations:** ^1^ Department of Public and Occupational Health, Amsterdam UMC, Amsterdam Public Health Research Institute Vrije Universiteit Amsterdam Amsterdam The Netherlands; ^2^ PATIENT+ Den Haag The Netherlands

**Keywords:** breast cancer, cocreation, health literacy, personalized information, risk communication, shared decision‐making

## Abstract

**Background:**

To support patients in shared decision‐making about treatment options, patient decision aids (PtDAs) usually provide benefit/harm information and value clarification methods (VCMs). Recently, personalized risk information from prediction models is also being integrated into PtDAs. This study aimed to design decision‐relevant information (i.e., personalized survival rates, harm information and VCMs) about adjuvant breast cancer treatment in cocreation with patients, in a way that suits their needs and is easily understandable.

**Methods:**

Three cocreation sessions with breast cancer patients (*N* = 7–10; of whom *N* = 5 low health literate) were performed. Participants completed creative assignments and evaluated prototypes of benefit/harm information and VCMs. Prototypes were further explored through user testing with patients (*N* = 10) and healthcare providers (*N* = 10). The researchers interpreted the collected data, for example, creative and homework assignments, and participants' presentations, to identify key themes. User tests were transcribed and analysed using ATLAS.ti to assess the understanding of the prototypes.

**Results:**

Important information needs were: (a) need for overview/structure of information directly after diagnosis and; (b) need for transparent benefit/harm information for all treatment options, including detailed harm information. Regarding VCMs, patients stressed the importance of a summary/conclusion. A bar graph seemed the most appropriate way of displaying personalized survival rates; the impact of most other formats was perceived as too distressful. The concept of ‘personalization’ was not understood by multiple patients.

**Conclusions:**

A PtDA about adjuvant breast cancer treatment should provide patients with an overview of the steps and treatment options, with layers for detailed information. Transparent information about the likelihood of benefits and harm should be provided. Given the current lack of information on the likelihood of side effects/late effects, efforts should be made to collect and share these data with patients. Further quantitative studies are needed to validate the results and to investigate how the concept of ‘personalization’ can be communicated.

**Patient or Public Contribution:**

Ten breast cancer patients participated in three cocreation sessions to develop decision‐relevant information. Subsequent user testing included 10 patients. The Dutch Breast Cancer Association (BVN) was involved as an advisor in the general study design.

## 1 INTRODUCTION

Decisions about treatment options often involve complex trade‐offs between benefits and harms for the individual patient. The principles of shared decision‐making (SDM), where patients and health professionals share information and patients are supported to weigh options to achieve informed preferences,^1^ are nowadays seen as the ideal when such decisions are made, especially when decisions are thought to be preference‐sensitive.^2^ An example of a preference‐sensitive decision about treatment options is the decision concerning adjuvant therapy after surgery for breast cancer patients. Adjuvant therapy can reduce the risk of metastasis and recurrence, improving life expectancy,^3^ but usually also comes with harms, such as side effects and lower quality of life.^4^ Decision‐support tools such as patient decision aids (PtDAs) and Option Grids can be helpful, providing benefit/harm information about treatment options and value clarification methods (VCMs).^5^, ^6^ Recently, personalized risk information from prediction models is also increasingly being integrated into PtDAs.^7^ However, processing and using the information in decision‐support tools is not easy, especially for patients with lower health literacy (HL) and/or numeracy.^8^ Therefore, it is important to design decision‐relevant information that suits the needs of patients and at the same time is clear for patients with diverse levels of HL/numeracy.^8^, ^9^

The WHO definition of HL is: ‘the cognitive and social skills which determine the motivation and ability of individuals to gain access to, understand, and use information in ways which promote and maintain good health’^10^ In Europe, 47% of the population has too few skills to understand and use health information correctly to make health‐related decisions.^11^ In the United States, 36% of adults have basic or below‐basic HL.^12^ Numeracy, or health numeracy, refers to skills required to understand and use quantitative health information, perform basic computations and compare magnitudes.^13^, ^14^ Numerical presentation formats and the use of visualizations have been studied in multiple risk communication experiments,^15^, ^16^, ^17^ but due to variances in study design and choice presented, it is difficult to draw conclusions about the best communication format.^18^ A review of the International Patient Decision Aid Standards (IPDAS) Collaboration provides some overarching best practices,^19^ for example, using the same denominator when two or more chances need to be compared.^7^

A complicating factor in communicating personalized survival rates predicted by a prediction model is that instead of population‐based estimates, personalized estimates based on an underlying algorithm are used. The personalization applies to the specific situation of the patient, for example, age and disease characteristics like tumour size and lymph node status, and therefore can be regarded as more personally relevant. According to information processing theories, this personalization can increase people's information processing motivation.^20^, ^21^ A recent study indeed found that personalized risks of cancer treatment side effects from a prediction model were perceived as more personally relevant than generic risks.^22^ However, this study also demonstrated that verbal descriptions of personalized risks were associated with higher risk perceptions, higher perceptions of certainty and lower perceptions of accuracy, compared to a format where verbal descriptions were accompanied by numbers. These differences were not found with generic, population‐based risks.^22^ So it may be that personalization makes risk information more complex to understand, for example, because the underlying principle of the algorithm is not fully clear. As the possibilities for creating personalized risk information are ever‐increasing, it is important to gain more insight into how this information should be presented^7^ and integrated with other decision‐relevant information available in PtDAs.

Collaboration with patients, for example using user‐centred design,^23^ is increasingly considered important when developing tools containing decision‐relevant information. The user‐centred design framework has been applied in several projects that developed PtDAs^24^, ^25^ and is also recommended by the IPDAS Collaboration.^26^ In this study, we used cocreation to gain insight into patients' perspectives. By ‘cocreation’ we mean actively involving the target audience, here women with breast cancer, in the development process of decision‐relevant information about adjuvant breast cancer treatment. We see the target audience as ‘experts of their experience’.^27^ By using various creative assignments and exercises, we supported patients to express their ideas and take part in the development process. These methods are also suitable for participants who are less verbally oriented and for expressing more latent thoughts and feelings, thereby enriching data collection.

This study is part of the project entitled ‘Personalized decision support systems in breast cancer care: integrating prediction modelling with user‐centred research’, which explores the integration of personalized estimates with information in PtDAs. Details about the prediction modelling are described elsewhere.^28^ The current study focuses on how the total package of decision‐relevant information (i.e., personalized survival estimates from a prediction model, other benefit/harm information and VCMs) can be communicated in a way that suits patients' needs and is easily understandable for patients with varying HL/numeracy levels.

This paper describes: (1) the process of information development, using cocreation; and (2) the key findings that emerged during this process.

## 2 MATERIALS AND METHODS

### 2.1 Study design

**Figure 1:**
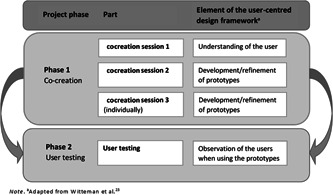
An overview of the project phases

This study used a qualitative approach consisting of two phases: cocreation (Phase 1) and user testing in which generated ideas and prototypes were further explored with patients and healthcare providers (Phase 2). An overview of phases with corresponding elements of the user‐centred design framework is shown in Figure 1. Table 1 provides a summary of the objectives, methods and results/key insights derived.

**Table 1:** Summary of the objectives, methods and results and key insights

Part, participants and researchers	Objective	Method	Results and key insights
*Phase 1*			
Cocreation	To gain insight into patients' information needs	Discussing information needs in groups by using a general treatment trajectory timeline	Information needs:
Session 1			Quick overview of the steps and treatment options at the beginning of the trajectory, with detailed information later on. (Numerical) information about survival, recurrence, side effects and late effects for the treatment options. Information about the time available to consider adjuvant treatment options. Information about healthcare providers, psychological support and follow‐up care. Information about lifestyle and alternative treatments, for example, nutrition and hyperbaric oxygen. Information and attention for spouse and children, that is, the PtDA itself but also options for psychological support.
Patients (*n* = 10)			
Researchers (O. D., J. B., I. S., M. B.)			
	To explore the ideal decision support tool	Sketching and presenting the ideal decision support tool in small groups	Key aspects regarding content:
			Information about applicable options with their benefits and harms. Information about cohesion and potential combination of multiple treatments. A treatment plan with a timeframe. An exercise to discover values/preferences. Emphasizing freedom of choice Overview of resources and additional information.
			Key aspects regarding design:
			An app suitable for a mobile phone. Possibility to read only the information you want. Pictures/animations. Possibility to make notes.
Cocreation	To gain insight into how the benefits and harms of the treatment options should be presented	Creating a poster to show what the presentation of benefits and harms in a decision support tool should look like	Important aspects:
Session 2			
Patients (*n* = 8)			Only showing relevant options. Information about treatment options and their duration. Numbers on survival rates. Information about side effects and late effects with the possibility for more information/descriptions. Information about solutions/medication for side effects. Pictures. Emphasizing freedom of choice.
Researchers (O. D., J. B., I. S., M. B.)			
	To gain insight into how to visualize the survival rates	Assessing six survival rate visualizations for preferences and subjective and objective comprehension	Visualizing personalized survival rates:
			Represent different treatments with different colours. No use of additional pictures. Preference for bar graph. For the less educated, the icon array may be most suitable.
Cocreation	To gain insight into the best way to design a value clarification method	Individual reflection on seven value clarification methods	Important aspects:
Session 3 (individually)			Simple language/easy to understand. Balanced exercise (not too difficult or too easy) to stimulate reflection. A mix between choosing from a set of predefined statements and own input. Use of pictures/photos. A design that fits the personal character of a value clarification method. Inclusion of a summary/conclusion.
Patients (*n* = 7)			
Researchers (J. B., O. D.)			
	To gain insight into how to visualize the likelihood of side effects	Individual reflection on six visualizations of the likelihood of side effects	Mixed results with four preferred prototypes; two horizontal bars, an icon array and a bar graph.
*Phase 2*			
User testing	To explore the comprehension of a summary table of the benefits and harms (patients only)	Interviewing patients using the summary table	Important aspects:
Patients (*n* = 10)			An overview of options and outcomes is useful. Include numbers. Add descriptions about side effects and late effects. Emphasize that late effects differ from person to person.
Healthcare providers (*n* = 10)			

Researchers (W. B., I. S., O. D.)	To evaluate the adapted survival rate visualizations (patients and providers)	Answering questions about: First impression, gist of the information, risk perception, uncertainty, personalization and preference	Bar graphs are preferred by both patients and healthcare providers; the advantage of the icon arrays is that they feel more personal.
	To evaluate the adapted likelihood of sideeffect visualizations (patients and providers)	Same as in point 2	The horizontal bar without a legend was preferred by both patients and healthcare providers.

Abbreviation: PtDA, patient decision aid.

The study was exempted from extensive review by the medical research ethics committee of Amsterdam UMC, location VUmc (FWA00017598) in accordance with local regulatory guidelines/standards for human subjects' protection in the Netherlands (Medical Research Involving Human Subjects Act). Both patients and healthcare providers provided informed consent.

### 2.2 Participants

We invited participants (both patients and healthcare providers) who had indicated in a previous questionnaire study to be interested in further research into the subject of breast cancer care. All patients were diagnosed with breast cancer between 2009 and 2019 and had undergone surgery. Ten of them participated in the cocreation sessions and 10 in the user testing. In the user testing also 10 healthcare providers participated. Patients completed a questionnaire assessing age, educational level, numeracy (Dutch version of the Subjective Numeracy Scale),^29^, ^30^ and HL (Dutch version of the Functional Communicative and Critical Health Literacy Scales).^31^, ^32^

### 2.3 Procedure and measures

#### 2.3.1 Phase 1: Cocreation

During the first cocreation session, participants were asked to indicate important decision moments on a timeline, their feelings and experiences at those moments, and what information they would have wanted at what time. Participants also sketched their ideal PtDA for adjuvant treatment, including benefit/harm information in probability form. Before the session, participants filled out a sensitizing booklet (Material S1), meant to prepare participants for participatory sessions by activating memories and experiences about a certain topic,^27^ in this case patients' breast cancer treatment processes.

In the second session, participants created a poster in a group assignment, displaying benefit/harm information they considered relevant in decision‐making. Participants then evaluated six prototypes of visualizations of personalized survival rates, designed based on the outcomes of Session 1 and the risk communication literature (Material S2). Participants made positive and negative comments on the prototypes and filled in four open‐ended comprehension questions, for example, ‘Do you think the benefit of the anti‐hormone treatment in terms of extra survival is big or small?’^15^, ^33^ and ‘How many out of 100 people would be alive after 10 years if they didn't take additional treatment?’.^16^, ^33^ Prototype preference was also assessed. To familiarize participants with PtDAs, they evaluated an existing Dutch PtDA on the adjuvant treatment of breast cancer at home before the session.

In the third session, participants reflected on seven VCM prototypes, designed based on the outcomes of the previous sessions and VCMs literature. Participants also reflected on six visualizations of the likelihood of side effects. Fatigue and nausea were used as case examples. Unfortunately, numerical information about the likelihood of side effects was not easily available in the Netherlands. A quick scan of the literature and medical web pages was performed, but merging this information was not possible due to variations in research methods.^34^ To estimate probabilities of fatigue and nausea, we used one self‐reported questionnaire study among 404 Dutch patients.^35^ Participants indicated positive and negative aspects about the VCMs and side effect visualizations, and answered questions per side effect visualization, that is, ‘How likely do you think it is that you will experience this side effect?’, using a 7‐point scale (1 = not at all likely; 7 = very likely)^36^ and ‘How concerned are you about getting the fatigue side effect?’, using a 7‐point scale (1 = not at all concerned; 7 = very concerned).^37^ We used standard probing questions to further explore patients' answers. Due to Covid‐19 restrictions, this session could not take place physically, so assignments were sent as homework (Material S3) and participants were interviewed by phone.

#### 2.3.2 Phase 2: User testing

**Figure 2:**
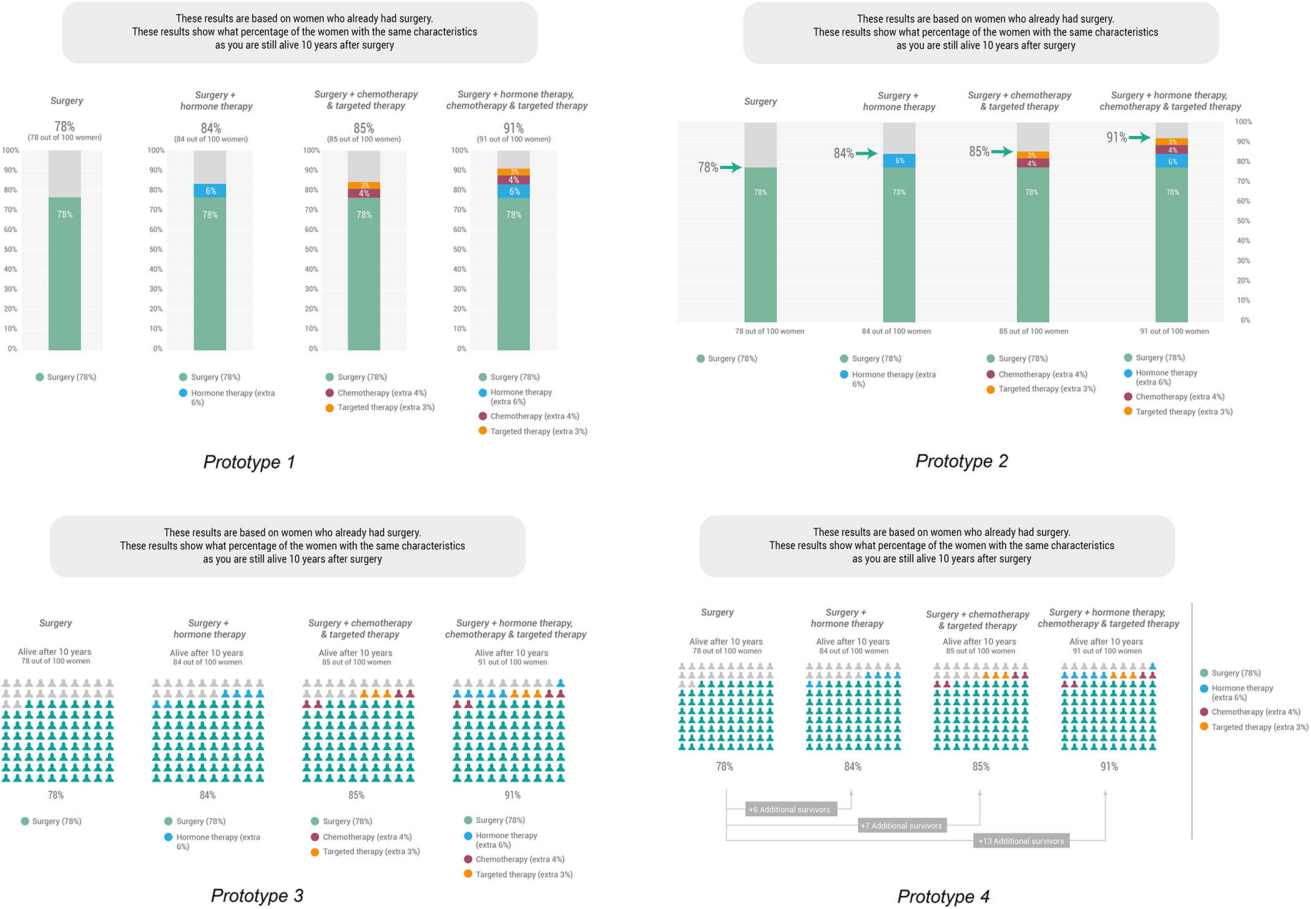
Prototypes of visualizations of personalized survival rates

**Figure 3:**
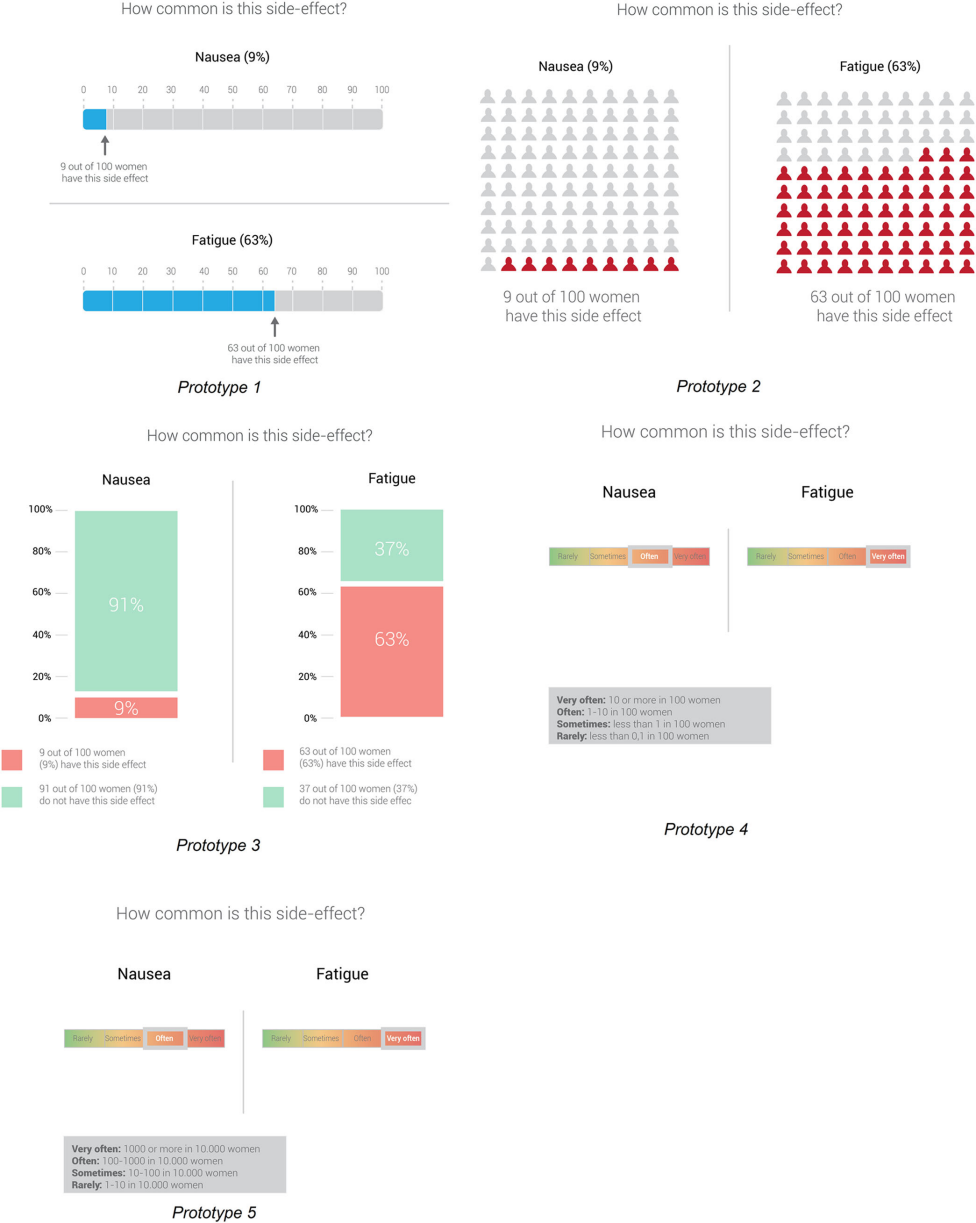
Prototypes of visualizations for the likelihood of side effects

Based on the findings of the cocreation sessions, various decision‐relevant information elements were further developed: (1) a summary table displaying benefit/harm information related to adjuvant treatment options, (2) four prototypes of visualizations of personalized survival rates from a prediction model (Figure 2) and (3) five prototypes for visualizing the likelihood of side effects (Figure 3). Tests were conducted by phone, due to Covid‐19 restrictions.

Patients received a link to an existing Dutch online PtDA, which was the same PtDA as in the cocreation phase, but now included a newly developed summary table. Patients and healthcare providers received prototypes of four personalized survival rate visualizations and five visualizations of the likelihood of side effects. Healthcare providers did not evaluate the existing PtDA, due to the limited time they had available. User testing started with sociodemographic questions. For the patients, the test continued with questions about the PtDA, for example, first impression, perceived helpfulness in decision‐making and perceived gist and verbatim meaning of information in the new summary table.^38^, ^39^ Subsequently, patients and healthcare providers answered questions about the visualizations, for example, first impression, gist understanding of information, risk perception, uncertainty of the numbers, personalization and preferred visualization format.^15^, ^16^, ^22^, ^33^, ^40^

### 2.4 Analysis

The data collected during the cocreation sessions were diverse, for example, audio‐recordings of discussions between participants, materials created and presentations of the participants. The data obtained are the results of a group process and are therefore analysed in a different way than results obtained from individual data collection methods. To guide our analysis of the cocreation sessions, we used the Data‐Information‐Knowledge scheme as used by Sanders and Stappers.^27^ After each session, one researcher summarized the data and this was discussed in consultation with the other researchers. Together, we moved from data to information by interpreting the data. The following step in the analysis process is knowledge, which means identifying patterns in the interpretations. The researchers together derived the main topics from the sessions. Since designing prototypes is an iterative process,^41^ insights from one cocreation session were incorporated into designing the prototypes for the next cocreation session/user testing. As recommended in this type of research, the development of the various prototypes was documented, as were the insights gained during the cocreation sessions.^27^ The homework assignments and questionnaires were collected and analysed as well. User tests were transcribed literally and analysed inductively using ATLAS.ti 8.^42^ Information from the transcripts on the same topic was categorized. Based on the analysis, it was assessed which prototypes were better and which were less well understood and appreciated. Also, the elements contributing to the understanding of the prototypes were identified.

## 3 RESULTS

### 3.1 Participant characteristics

The 10 cocreation participants lived in different parts of the Netherlands and were treated in different hospitals. Participants' characteristics of both the cocreation sessions and the user test are displayed in Table 2.

**Table 2:** Characteristics of the participants in the cocreation sessions and the user testing

	Group session 1 (*n* = 10)	Group session 2 (*n* = 8)	Individual session 3 (*n* = 7)	User testing (*n* = 10)
Demographics				
Age (years), median	53.5	48.5	50.0	54.0
(Youngest–oldest)	(46–68)	(46–68)	(46–68)	(44–68)
Education level				
Middle	4 (40%)	3 (37.5%)	4 (57.1%)	1 (11.1%)
High	6 (60%)	5 (62.5%)	3 (42.9%)	8 (88.9%)
Health literacy				
FCCHL^a^—high	5 (50%)	3 (37.5%)	3 (42.9%)	5 (55.6%)
FCCHL—low	5 (50%)	5 (62.5%)	4 (57.1%)	4 (44.4%)
Numeracy				
SNS,^b^ median (IQR)	4.6 (4.1–5.3)	5.0 (4.0–5.5)	4.6 (3.9–5.2)	4.4 (3.9–4.8)
(Range: 1–6)	(*n* = 9)	(*n* = 7)	(*n* = 6)	(*n* = 9)

Abbreviations: FCCHL, Functional Communicative and Critical Health Literacy Scales; IQR, interquartile range; SNS, Subjective Numeracy Scale.

^a^ Functional Communicative and Critical Health Literacy Scales. Fourteen items are measured on a 4‐point scale and the total score is the average, ranging from 1 (low HL) to 4 (high HL),^31^ with 3 points or less being defined as having low HL.^32^

^b^ Subjective Numeracy Scale. Items are measured on a 6‐point scale. The total score is the average of all items, ranging from 1 (low numeracy) to 6 (high numeracy).^29^, ^30^

The user tests took about 1 h for patients (*N* = 10) and half an hour for healthcare providers (*N* = 10). Of the healthcare providers, 30% were nurse specialists, 30% internist oncologists and 40% (oncology) surgeons. The period they had been working in their current specialization varied from 1.2 to 25 years (*M* = 11.4 and SD = 8.0).

### 3.2 Main findings

The following section describes the main topics that emerged from the cocreation sessions and user testing. Quotes from the individual user testing interviews are used to illustrate the topics.

#### 3.2.1 General information needs: Overview and transparency

Two important points emerged in the exploration of information needs. First, there was a need for an overview and structure. For example, an overview highlighting relevant information with more detailed information when this becomes relevant throughout the patient journey. Second, participants stressed a need for transparent information about benefits and harms related to the relevant options, including the option not to have adjuvant therapy. Linked to these information needs, key aspects that participants wanted to see were: typical treatment plans with accompanying timeframes, a glossary of medical terms, and overviews of providers and other types of support (e.g., lifestyle and psychological support, contact with fellow sufferers, support in second opinions). Another recurring aspect mentioned was that the information provided should apply to the patients' situation to avoid confusion, for example, only display treatment options that apply to the specific patient. Another wish was an exercise to discover what is important to the individual, that is, a VCM.

*Women (44 years, high HL, interview 7) “What is very nice here [the PtDA], you fill in what type of cancer you have and then it starts running so to say. Normally, you have to read a lot of information that is also scary to read, that you don't want to read before you end up in the right place.”*

#### 3.2.2 Benefit/harm information: Need for numerical survival rates and detailed sideeffect information

Participants only wanted benefit/harm information relating to the options relevant to them. Furthermore, numerical survival rates were seen as important, as was more information about the side effects and late effects of adjuvant treatment and the likelihood of them occurring. Participants expressed a need to see a more detailed description of side effects, as well as potential solutions/medications to deal with them. Many participants, both in the cocreation sessions and in the user testing, indicated that information on side effects and late effects had been too limited when discussing adjuvant treatment options with their healthcare provider.

*Women (52 years, high HL, interview 6) “What I found very important, and that is neatly stated here on the site [PtDA], are things like late consequences. It is very nice that this is stated here because that is not discussed in the hospital.”*

#### 3.2.3 Survival rate visualizations: Bar graph seems the most appropriate format

Concerning visualizations of personalized survival rates, we experimented with visualizations that tried to connect with women's everyday experiences and to design less abstract visualizations, for example, by relating survival rates to the expected number of birthdays or Christmas celebrations. However, most participants in the cocreation sessions did not think these visualizations were of added value and felt that they evoked negative feelings. Another prototype that evoked negative feelings was a visualization that explicitly showed the number of deaths in a separate bar graph. Regarding comprehension of the visualizations used in the cocreation sessions, the prototypes in which the different options were represented by different colours were best understood. Participants tended to prefer a visualization with a bar graph.

In the user testing, no differences in gist understanding were found between the prototypes shown. However, bar graphs (Figure 2, prototypes 1/2) were preferred by most patient participants, with a slight preference for the former. Icon arrays (Figure 2, prototypes 3/4) were less positively evaluated, mainly because the number of icons was experienced as intense. However, some patients indicated that the icons did show that you are not alone as a patient. Healthcare providers also preferred bar graphs to explain personalized survival rates to their patients. Some also preferred icon arrays because they were thought to make abstract information more personal.

*Women (56 years, low HL, interview 4) “Well wow, I think indeed. What is meant here [icon arrays]? I know it's the same data [as in the bar graph], only because of those icons it does become a bit confusing. My eyes are going from left to right through the diagram to see what it says.”*

*Women (48 years, unknown HL, interview 5) “You really see [in the icon array] that there are 100 and how big that group is. Whoever you happen to be, you are not alone, you just see that better.”*

#### 3.2.4 Likelihood of side effect visualizations: Need to come from standards from patient information leaflets to comprehensible risk communication

Concerning visualizations of the likelihood of side effects, formats with a horizontal bar and an ‘x out of 100’ format, instead of abstract percentages, were most appreciated. However, some participants appreciated the use of verbal labels and icon arrays. In order not to focus only on the negative side, others suggested also mentioning those who do not experience the side effects in the classical bar graph. However, when we experimented in the user testing with such a visualization that included more explicit background information^43^ (Figure 3, prototype 3), patients thought that this prototype contained too much redundant information. As in the cocreation session, the preferred format was the horizontal bar, indicating the number of women experiencing a side effect using an ‘x out of 100’ format (Figure 3, prototype 1). The prototypes with a legend (Figure 3, prototypes 4 and 5) were considered unclear because interpreting the legend required much cognitive effort. The classification in the legend was based on the recommendations for patients information leaflets.^44^ However, participants felt that the legend terms used, for example, ‘often’ and ‘sometimes’, did not reflect how they would apply these terms, for example, the term ‘often’ was used when 1–10 out of 100 women had this side effect and participants would not classify 1–10 out of the 100 as ‘often’ but as ‘sometimes’. Healthcare providers' preferences in user testing corresponded with those of patients in all aspects.

*Women (45 years, low HL, interview 1) “Well you can say often, but then you have to read below what that 1 to 10 out of 100 women… okay, 1 to 10… well that is… then you see that… no (…) no, it's a lot of effort.”*

*Women (44 years, high HL, interview 7) “With “very often” I think about half. And when I think of “often” I think of 40%, and “sometimes” I think of 10, and “rarely” is under 5. But 10 “very often” I find… I find “very often” nauseous 1 in 10 I don't think that is much.”*

#### 3.2.5 VCM: The importance of a summary/conclusion

The VCM was considered an important element by the participants in the cocreation sessions (also see Section 3.2.1). They thought it would prompt them to prioritize what is personally important. Many participants indicated that the VCM should encourage reflection, and they suggested the use of active elements, for example, selecting pictures/statements and moving them to the correct position, an appropriate level of difficulty so that you have to make an effort, and prioritizing statements rather than considering statements as equally important. Participants wanted to receive a summary or conclusion of their values after completing the VCM.

#### 3.2.6 PtDA overall positively evaluated, but suggestions to include numerical probabilities

In the user testing, patients' responses to the PtDA were generally positive. Aspects that were particularly appreciated were the tailoring of the PtDA to the type of breast cancer; the fact that information could be read at home, also by spouse and family and the information about side effects and late effects. Regarding the newly developed summary table showing the key benefit/harm information, patients appreciated the fact that this created one overview of options and their outcomes. To further improve the summary table, participants suggested not only describing treatment effects verbally, for example, large or small risk, but including numerical probabilities and adding information on what the side effects and late effects exactly entail.

*Women (68 years, high HL, interview 2) “Well, it [summary table] is all neatly in boxes what something is of course and how long it takes. And it is very clear. And I think if you find yourself in such a situation where you are ill, then it is … then you are often a bit chaotic. Then this very clearly shows the pros and cons and how long it takes when you choose something and all that kind of things.”*

#### 3.2.7 Personalization was not adequately understood

When patients in the user testing were asked to explain the concept of ‘personalization’, several participants did not understand this. Some mentioned that survival rates were personalized on aspects such as having children or not, while in reality, medical parameters were taken into account, for example, tumour size and lymph node status. Others thought the personalization only concerned breast cancer type. When asked to think of more characteristics, only a few were able to indicate characteristics like age, tumour grade, menopause and lymph node status. Some participants could not imagine at all what ‘personalization’ could mean. When asking healthcare providers how they would explain this concept to patients, they indicated that they would fill in the characteristics in the consultation room in the presence of the patient. They recommended explicitly mentioning some characteristics, to support understanding.

*Women (65 years, low HL, interview 10) “A good one [question]. Women with the same characteristics as me. Well… (laughs)… I have no idea.”*

## 4 DISCUSSION

This study aimed to design a total package of decision‐relevant information about adjuvant breast cancer treatment in cocreation with patients, in a way that suits their needs and is easily understandable. One important element of our study was the integration of personalized survival rates from a prediction model with other information considered relevant for SDM, such as information on the likelihood of side effects and VCMs. Important information needs identified were: (a) the need for an overview/structure of information directly after diagnosis; and (b) a need for transparent benefit/harm information for all adjuvant treatment options, including detailed harm information. Regarding VCMs, patients stressed the importance of a summary/conclusion. A bar graph seemed the most appropriate way of displaying personalized survival rates. The concept of ‘personalization’ was not understood by multiple patients.

An important identified information need was that patients wanted an overview with important steps and treatment options highlighted at the beginning of the trajectory, with options for detailed information later on. This is congruent with previous studies among prostate and breast cancer patients,^45^ including recent findings where women with breast cancer indicated the need to place treatment choices in the context of the complete treatment trajectory.^46^ However, our participants also said they needed more detailed information through layered options, a finding also demonstrated in a recent study.^47^ These findings may be related to a more underlying need throughout the patient decision journey, namely that of being able to cope with large amounts of information during an emotional period. Preventing being overwhelmed by information in the chaotic and confusing period directly after diagnosis was a topic that emerged multiple times during cocreation sessions and user testing. Participants emphasized that they did need decision‐relevant information, but only at the right time and in a manageable form.

Another important information need was the need for transparent benefit/harm information, for example, survival rates and numerical information about the likelihood of side effects and late effects, related to all options. This need for numerical information about side effects and late effects is in line with previous observations among prostate and breast cancer patients.^45^, ^46^, ^48^ But while the need is well‐known and the IPDAS criteria also describe its importance,^7^ not all PtDAs typically provide this benefit/harm information in numerical form. For this specific decision, an explanation could be that numerical information about the likelihood of side effects is simply not easily available. This is problematic, especially as patients so clearly indicate needing this information^46^, ^49^ and that they might need even more extensive information, for example, about what side effects can mean for them personally and what they can do about it. Another reason for not providing benefit/harm information in numerical form can be the assumption that patients do not understand these numbers. However, there is supporting evidence that including numerical information in PtDAs is helpful for patients as long as the right format is used.^7^ General principles about clear formats are known, but depending on the context some formats can be perceived as containing biased rather than balanced information.^7^ To meet patients' need for numerical information, more research into clear and balanced numerical formats in different contexts is desirable.

In the cocreation sessions, we experimented with visualizations of personalized survival rates that would have a more intuitive meaning, that is, that tried to connect with women's everyday experiences, compared to more classical visual formats. However, these prototypes were not appreciated. The preferred visualization according to both patients and healthcare providers was the more classical bar graph. This is consistent with risk communication literature stating that bar graphs are well suited for comparisons across groups.^7^ However, we also know that comprehension of such visualizations is often not thoroughly evaluated among patients with a low numeracy/Graph Literacy (GL).^50^ It might be that a bar graph is clear enough for patients and a more intuitive visualization is not needed. However, we should keep in mind that although we had lower HL participants in our sessions, we did not succeed in involving the ones with the lowest levels of education (and probably also lower levels of numeracy/GL). The advantage of icon arrays is that they represent numbers in a graphical way,^7^ which may be advantageous for those with lower numeracy/GL. In addition, an icon array might also carry more affective meaning, for example, that you are part of a group of patients. Whether this affective meaning is helpful or too confrontational in the period after diagnosis needs further investigation. It is also important to consider the number of options presented, as four options displayed in icon arrays may cause more cognitive load compared to four options displayed in bar graphs.^51^ Therefore, depending on the number of options displayed in a PtDA, icon arrays or bar graphs may be more suitable.

Patients in our study recommended including opportunities to reflect on their values in a PtDA as these values play a role in preference construction. This finding is congruent with SDM best practices where VCMs in decision support tools are recommended.^52^ A specific finding regarding the VCMs was that a summary/conclusion at the end of the VCMs they were provided with was lacking, which is a finding in line with previous literature in this field. A systematic review of VCMs showed that feedback was provided in less than a third of the included VCMs.^53^ Therefore, the inclusion of a summary/conclusion can be of added value in the further development of VCMs.

An important element in this study was the integration of personalized survival rates from a prediction model with other information considered relevant for SDM. It appeared that the concept of personalization was not easily understood by everyone. As addressed in the risk communication literature, the explanation of this concept should be further investigated to help patients understand its meaning, usefulness and limitations.^7^

### 4.1 Strengths and limitations

A strength of our study is our in‐depth user‐centred design approach; as recommended in the user‐centred design framework,^23^ we involved users in each of the key elements.^24^, ^25^ Of the 20 study participants, about half had low HL, as measured by the FCCHL. So although most participants had a medium or high educational level, we did manage to include participants with various HL levels. This is important when developing decision‐relevant information that is understood and used by patients with different HL levels.^8^, ^9^ While participants were enthusiastic about the sessions and eager to attend them, some were unable to attend all sessions due to low energy levels. However, we had taken dropouts into account and therefore managed to include at least seven participants per session.

During the cocreation sessions, we used a variety of creative exercises and assignments to stimulate participants to share thoughts and ideas. These types of assignments can yield insights that remain underexposed in a direct way of research, for example, because respondents are not—or less—aware of some thoughts or feelings. Moreover, this approach not only allows participants who can express themselves well verbally to share their thoughts and ideas but also the ones who have more difficulty doing so. This enriched data collection and the results will therefore probably also apply to a wider user group. However, according to the researchers' observations, participants belonging to the main ethnic minorities in the Netherlands were not well represented among the participants. To validate the results, more research among ethnic minorities is necessary. In addition, quantitative research is needed to validate the results in a larger and more representative user group. Unfortunately, due to Covid‐19, the third cocreation session could not take place physically, preventing some of the benefits of cocreation sessions from taking place, for example, observing participants as they work with materials and interaction between participants.

A final potential limitation was that the user testing mainly contained subjective questions regarding comprehension, preferences and opinions, which do not always correspond to objective comprehension and the potential additional benefit that visualizations can have.^54^

## 5 CONCLUSION

To foster SDM, PtDAs about adjuvant breast cancer treatment should highlight the most relevant information for the individual patient in the emotional period directly after diagnosis, but at the same time provide layered options to all kinds of breast cancer‐related information, including for relatives/loved ones. Moreover, patients want access to transparent benefit/harm information, for example, survival rates and numerical information about the likelihood of side effects and late effects. Given the current lack of detailed information about side effects/late effects, efforts should be made to collect and share these data with patients, to support their trade‐off between benefits and harms.

## AUTHOR CONTRIBUTIONS

All authors made valuable contributions to the study. Inge S. van Strien‐Knippenberg and Olga C. Damman designed and developed the study, Marieke C. S. Boshuizen and Domino Determann provided feedback. Jasmijn de Boer created the information prototypes and the materials used in the cocreation sessions. Inge S. van Strien‐Knippenberg, Olga C. Damman, Jasmijn de Boer and Marieke C. S. Boshuizen conducted the first and second cocreation sessions. Jasmijn de Boer and Olga C. Damman were responsible for the interviews in the third cocreation session. Olga C. Damman and Inge S. van Strien‐Knippenberg were responsible for the user testing and analysis. Inge S. van Strien‐Knippenberg drafted the manuscript and Olga C. Damman, Marieke C. S. Boshuizen, Domino Determann and Jasmijn H. de Boer critically reviewed the manuscript.

## CONFLICTS OF INTEREST

Marieke C. S. Boshuizen and Domino Determann were working at ‘PATIENT+’ at the time of the study. PATIENT+ is part of the consortium; it is a company that provides the patient decision support tool for breast cancer adjuvant therapy that was studied (https://patientplus.info/).

## Supporting information

Supporting information.

Supporting information.

Supporting information.

## Data Availability

Raw data of the cocreation sessions are not shared, given the privacy of participants and ethical restrictions. Raw data of the user testing are available on request from the corresponding author (in Dutch).
