# Supplementary material for: Cocreation with Dutch patients of decision‐relevant information to support shared decision‐making about adjuvant treatment in breast cancer care
Source: Health Expect. 2022 May 17;25(4):1664–77. doi: 10.1111/hex.13510 (PMC9327829; doi:10.1111/hex.13510)
Supplement: Supplementary file 1 — Supporting information. [file HEX-25--s003.pdf]

Diary

Preparation for the group interview

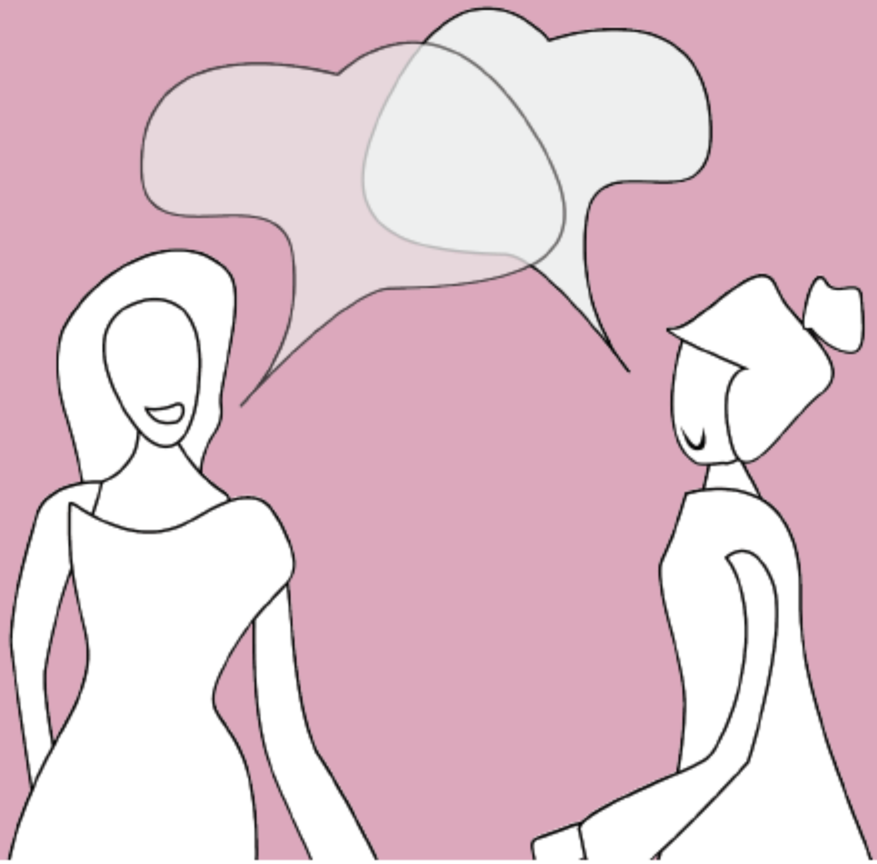

Introduction

- Thank you for participating in the group interview on Thursday, February 6st.
- This booklet consists of two assignments to be completed at home. There are no correct or incorrect answers. We are curious about your personal experiences during the treatment process.
- We kindly ask you to bring the completed booklet with you to the group interview on February 6st.

About me

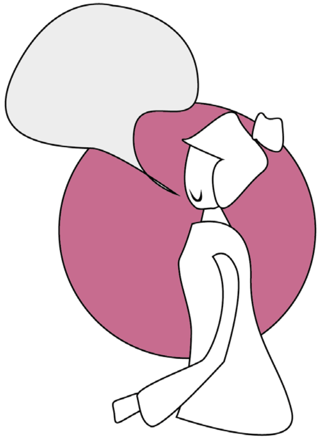

Name: ....

Residence: ....

Situation at home: ....
(e.g. single/married/children)

Hobbies: ....

Assignment 1

My timeline

Step 1: Please indicate the course of your treatment process on the timeline: which steps did you go through?

Discovery of breast cancer

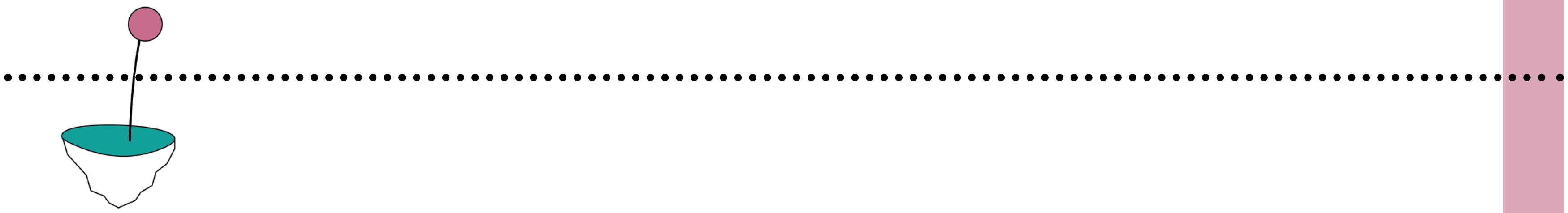

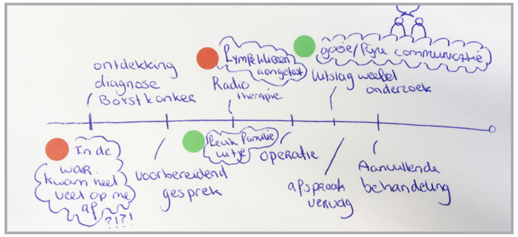

Example

Step 2: Mark 2 moments on the timeline where you experienced positive feelings. Use the green stickers and explain why you experienced this. Mark 2 moments on the timeline where you experienced negative feelings. Use the red stickers and explain why you experienced this..

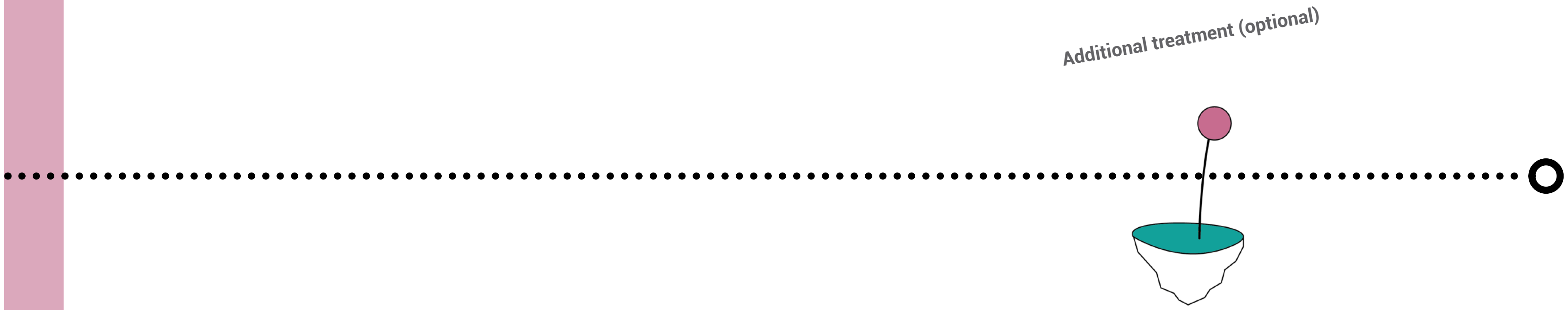

Assignment 2

This is important to me

On the next page, a list of general characteristics and values is shown.

Step 1: Circle the characteristics and values that apply most to you as a person (choose 5)

Step 2: For these 5 characteristics/values, please indicate whether they are important for you in the context of making a treatment decision. If so, indicate this with a plus (+). If not, indicate this with a minus (-).

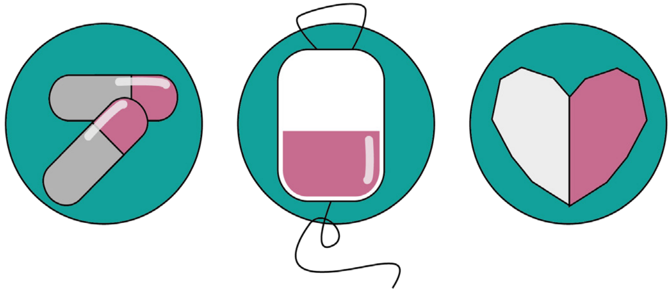

- *Ambition (strive for things in life)*
- *Autonomy (decide for yourself)*
- *Balance*
- *Helpfulness*
- *Proficiency (be good at something)*
- *Politeness*
- *Creativity*
- *Family*
- *Health*
- *Learning*
- *Charity (love for others / the environment)*
- *Nature*
- *Independence (can do things alone)*
- *Venturous (start new things)*
- *Personal growth (develop yourself)*
- *Pleasure*
- *Achievement*
- *Religion (faith)*
- *Being together*
- *Spirituality*
- *Spontaneity*
- *Status (have respect)*
- *Satisfaction (be satisfied)*
- *Challenge*
- *Responsibility*
- *Friendships*
- *Freedom*
- *Certainty (being sure)*
- *Self-discipline (strength to persist)*

Notes

This space can be used for additional notes:

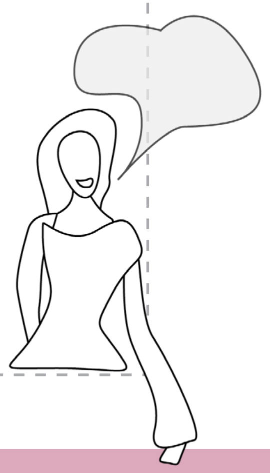

Thank you!

Thank you for sharing your knowledge and experiences!

Please remember to bring the booklet
