# Supplementary figures and images for: Cocreation with Dutch patients of decision‐relevant information to support shared decision‐making about adjuvant treatment in breast cancer care
Source: Health Expect. 2022 May 17;25(4):1664–77. doi: 10.1111/hex.13510 (PMC9327829; doi:10.1111/hex.13510)

1

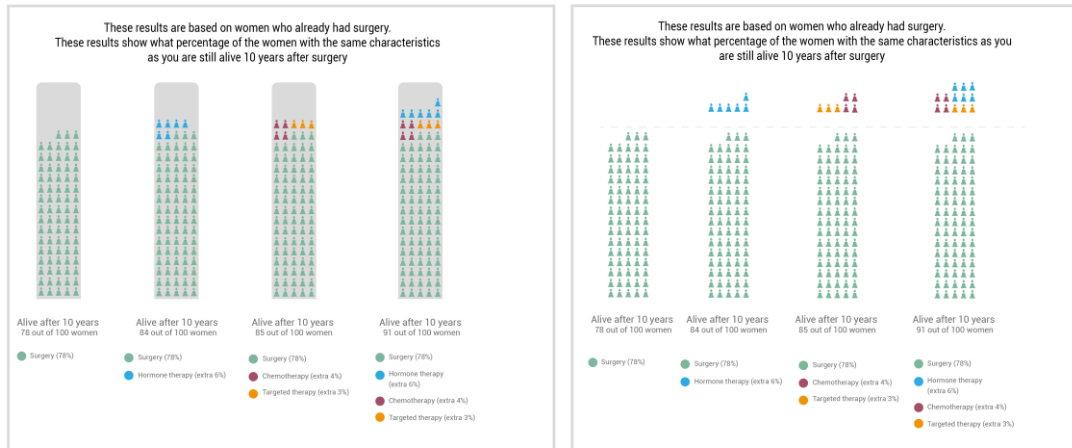

2

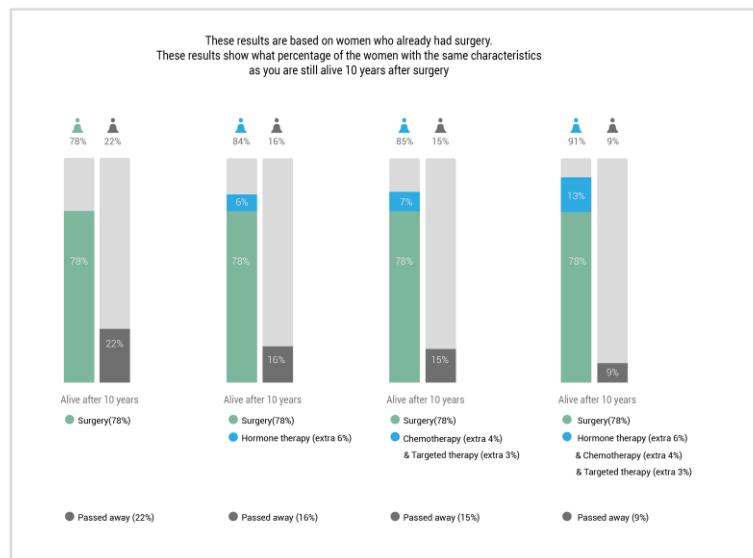

3

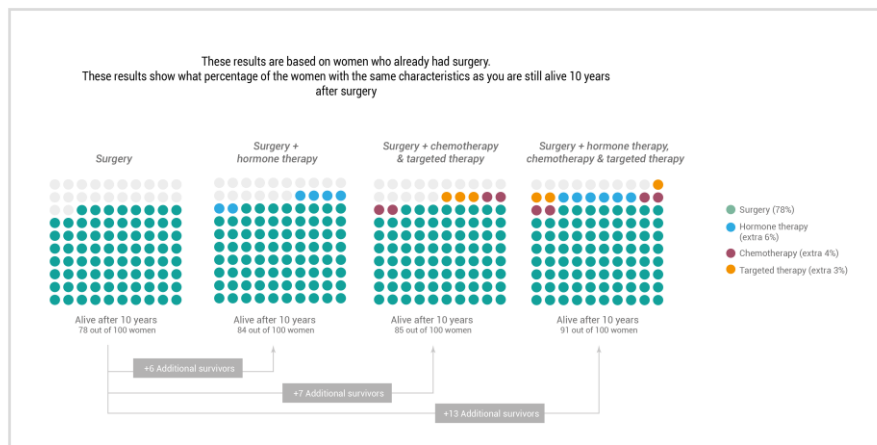

4

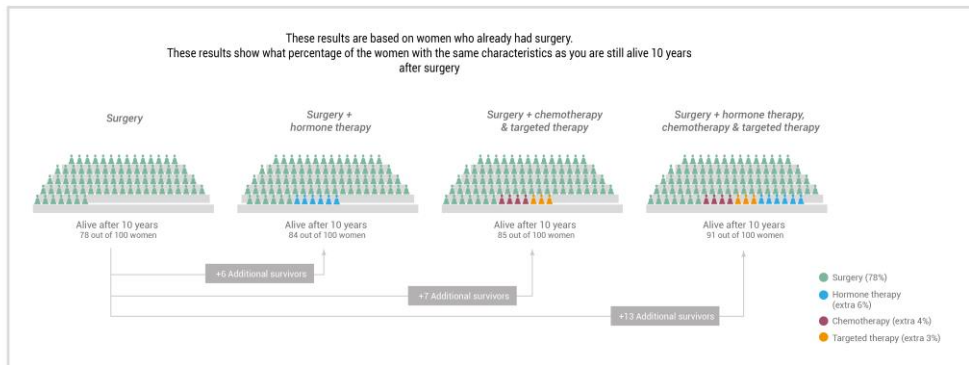

5

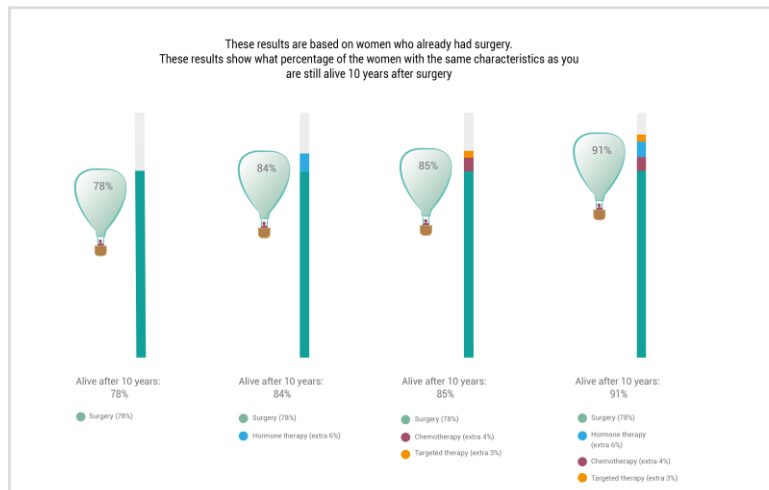

6

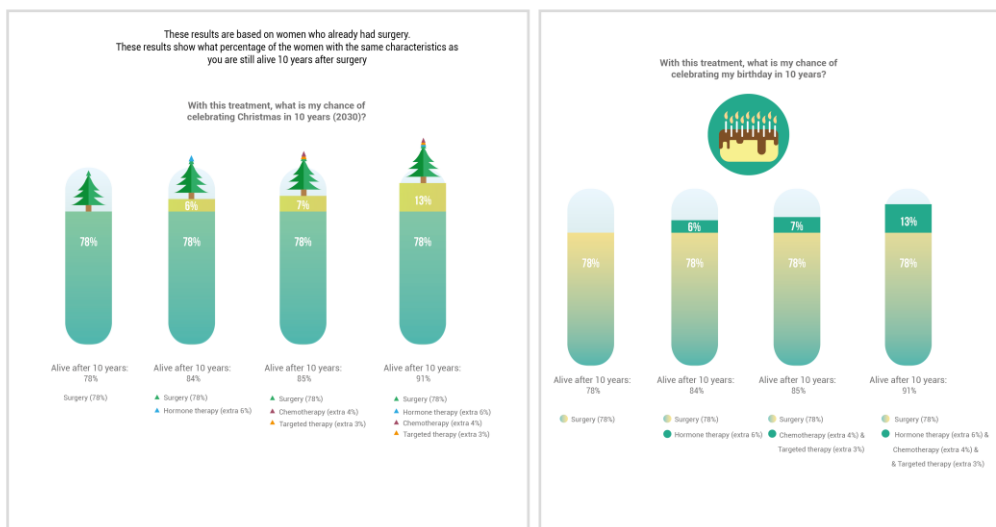

Supplement: Supplementary file 2 — Supporting information. [file HEX-25--s002.pdf]
