# Supplementary material for: Cocreation with Dutch patients of decision‐relevant information to support shared decision‐making about adjuvant treatment in breast cancer care
Source: Health Expect. 2022 May 17;25(4):1664–77. doi: 10.1111/hex.13510 (PMC9327829; doi:10.1111/hex.13510)
Supplement: Supplementary file 3 — Supporting information. [file HEX-25--s001.pdf]

Home assignment

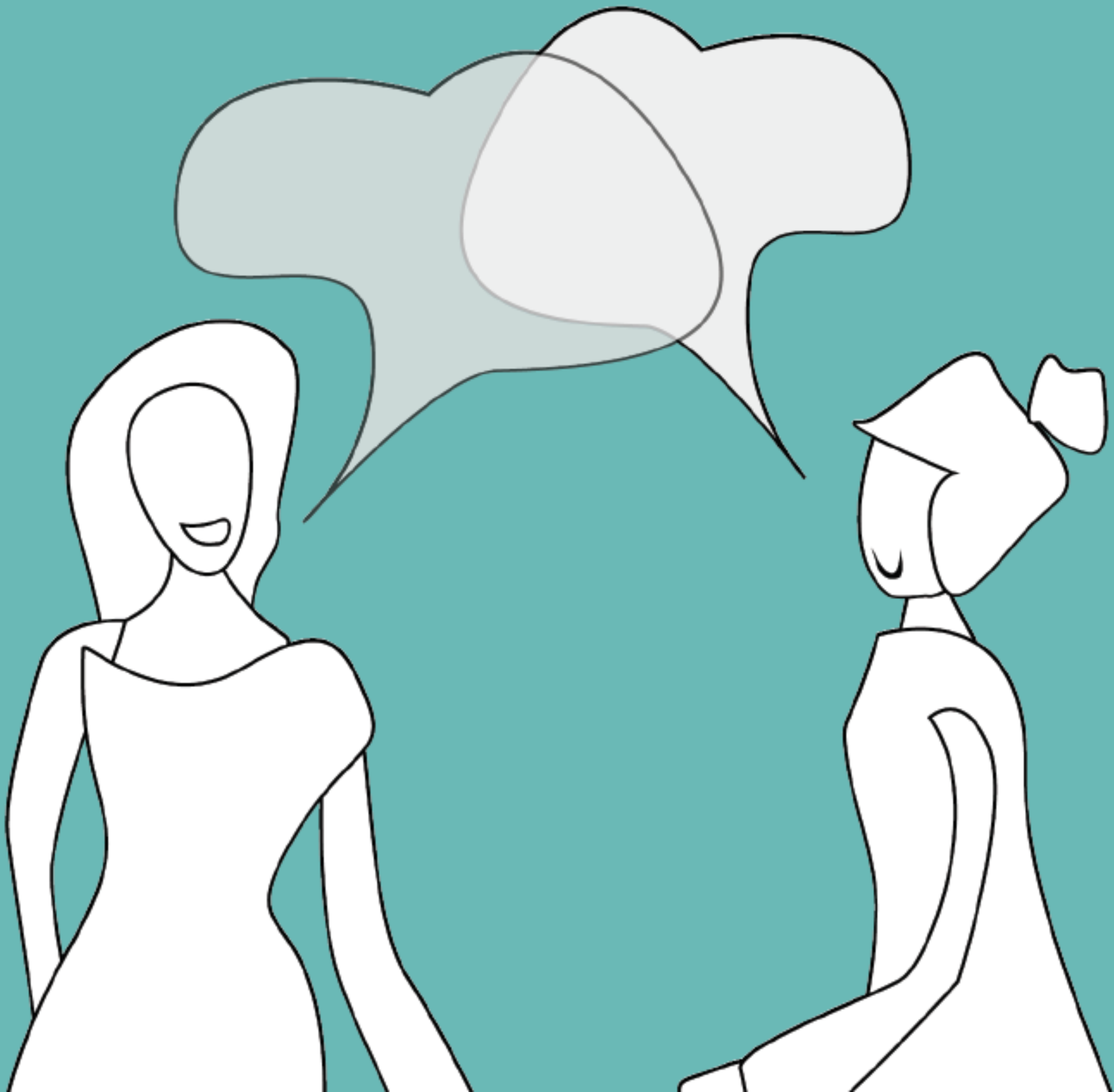

Assignment 1 and 2

The decision about undergoing one or more additional treatments after surgery is a personal choice.

To make it clear to the healthcare provider which things are important to you, various assignments can be used. You will now start with 2 of these assignments yourself. These assignments are now on paper, but it will eventually be an online assignment.

Assignment 1

You see yourself in the middle of several circles. You will also see various photos and icons on the next 2 pages.

The assignment:

- Choose some photos and icons that show what is important to you.
- Cut these out and stick them at the correct distance from the figure according to you
- Write down what you mean by the photo or icon
- Write or draw important things if you can't find a relevant picture or icon

This is what the healthcare provider needs to know about me:

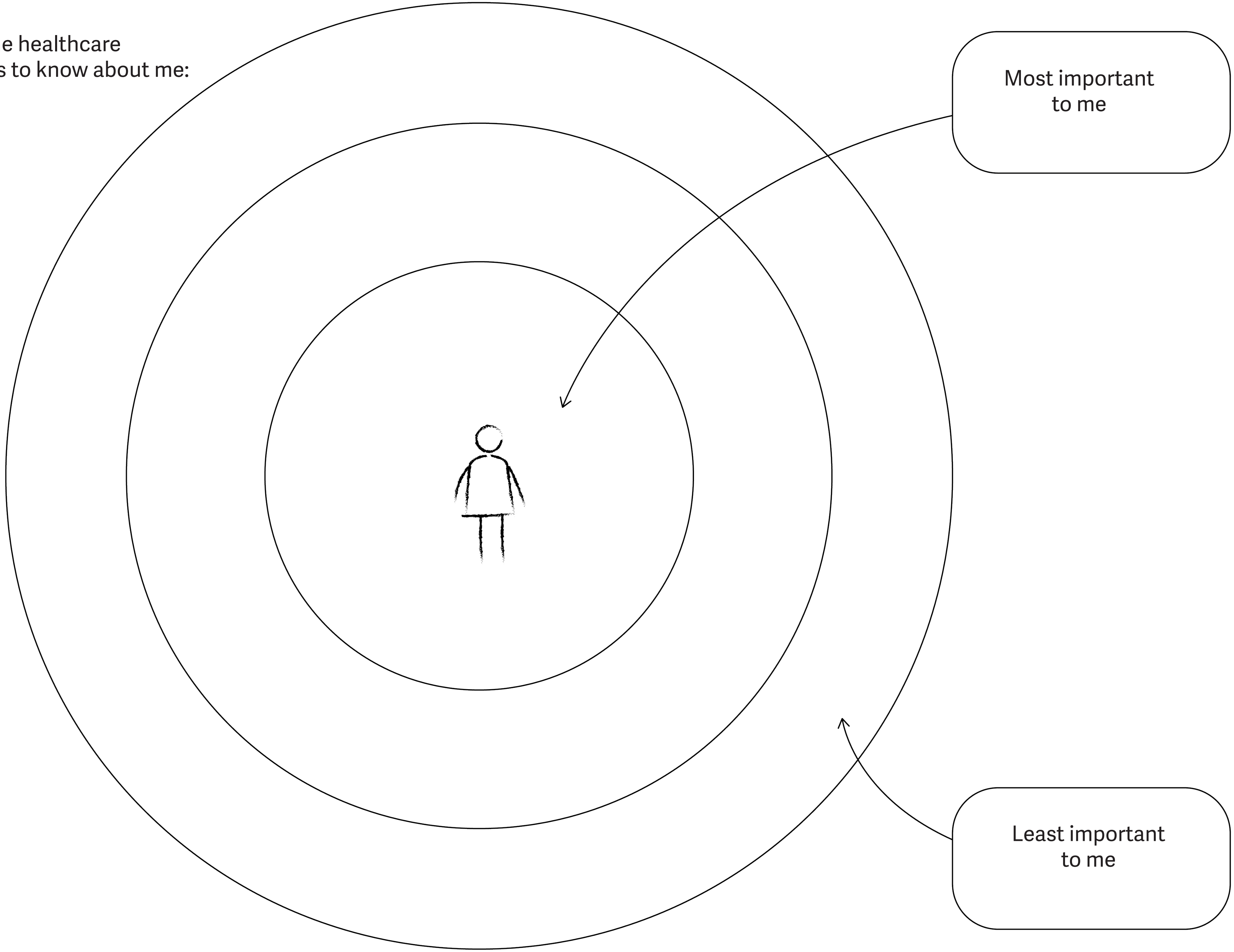

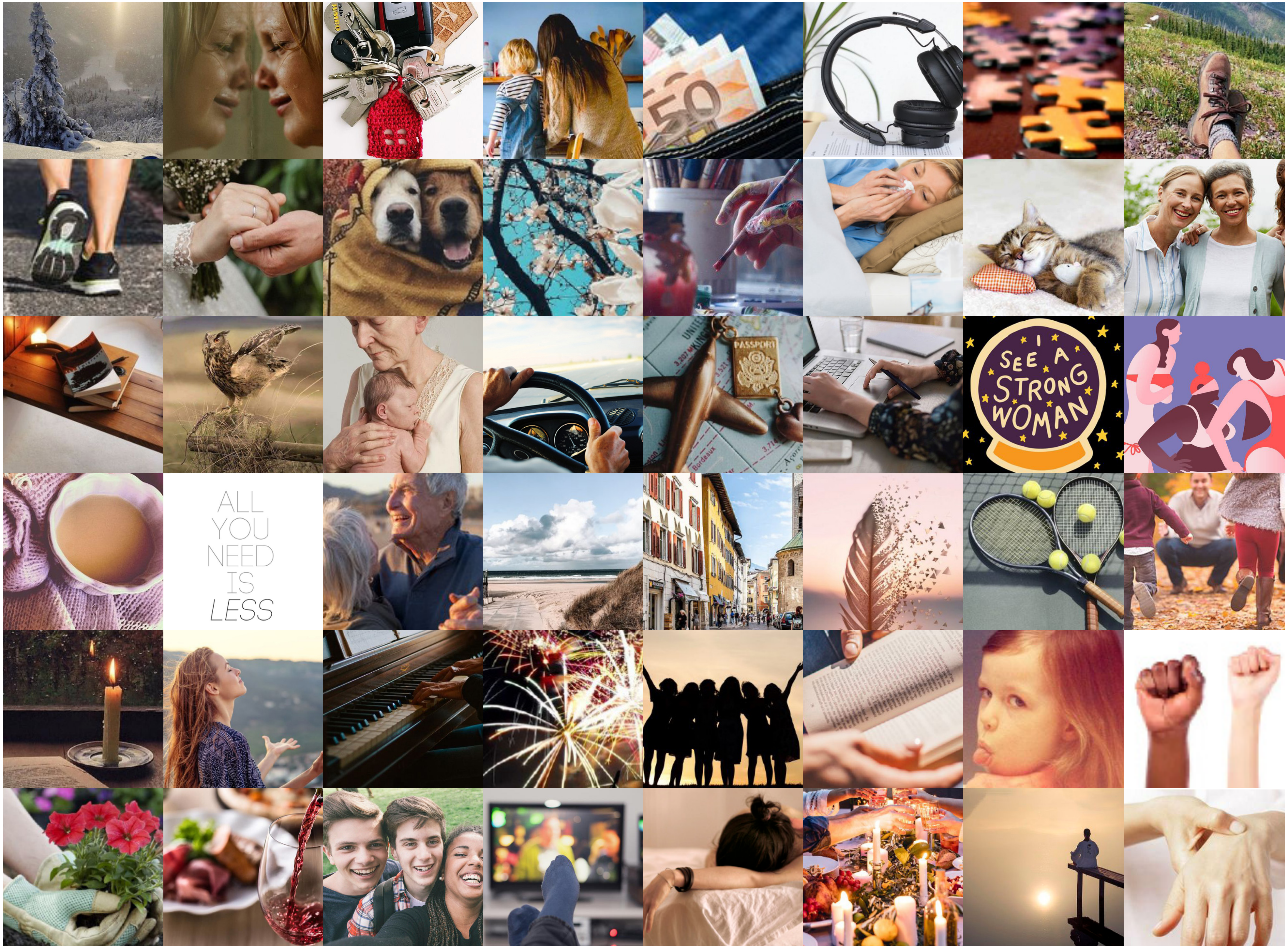

ALL YOU NEED IS LESS
I SEE A STRONG WOMAN
PASSPORT

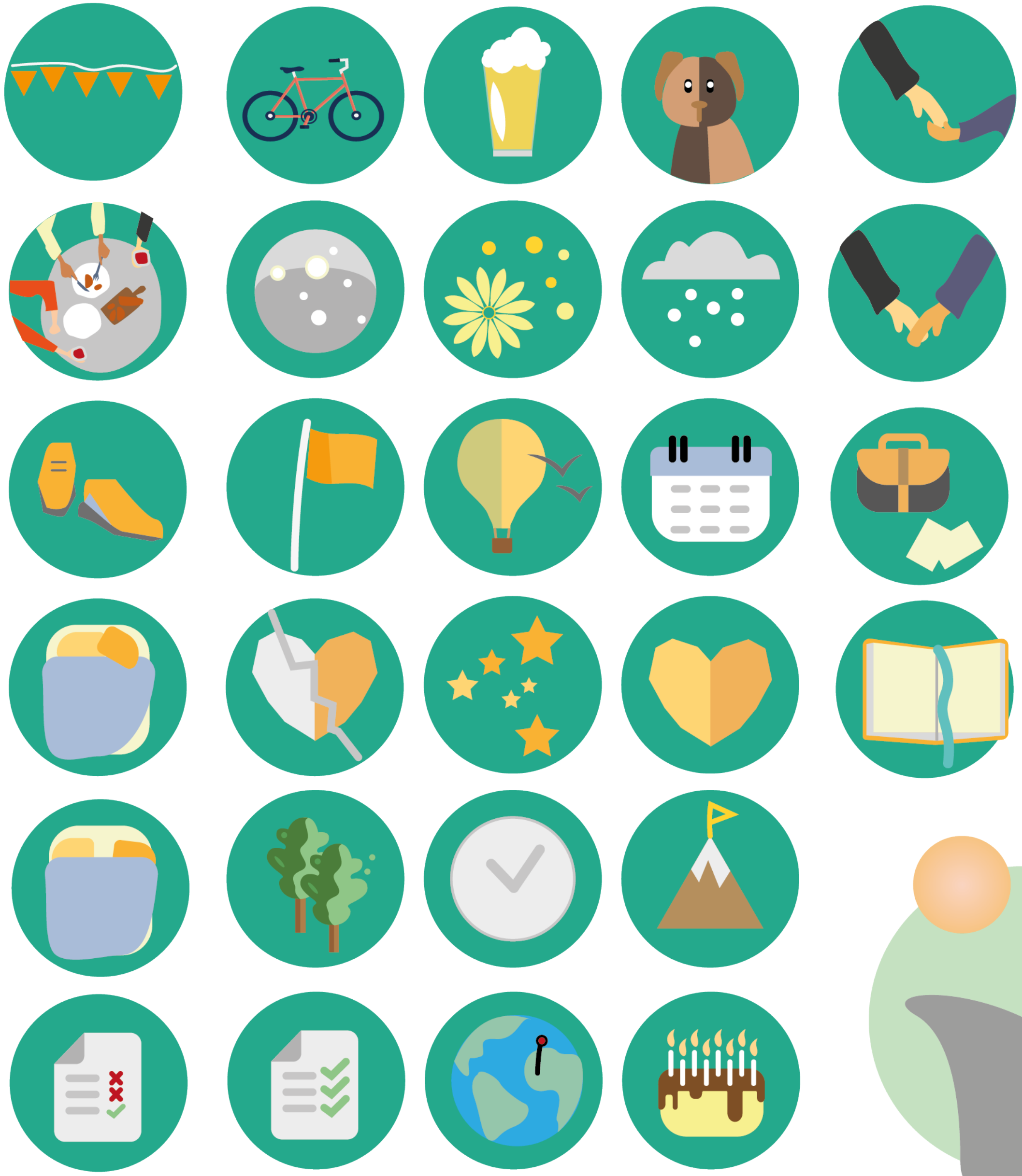
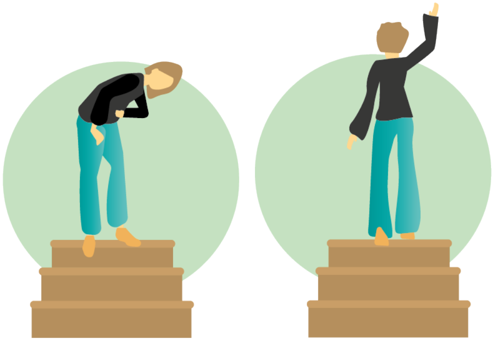
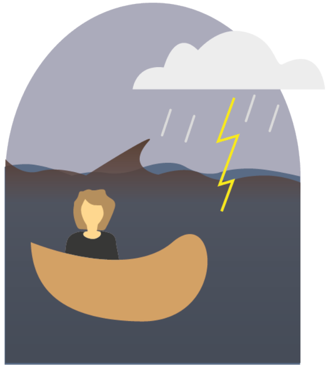
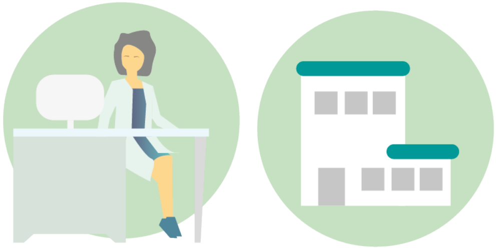
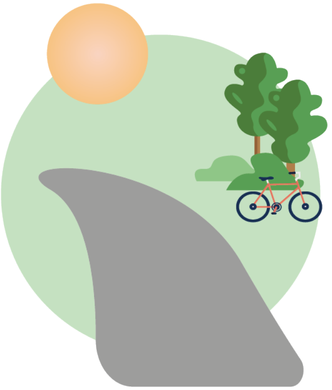
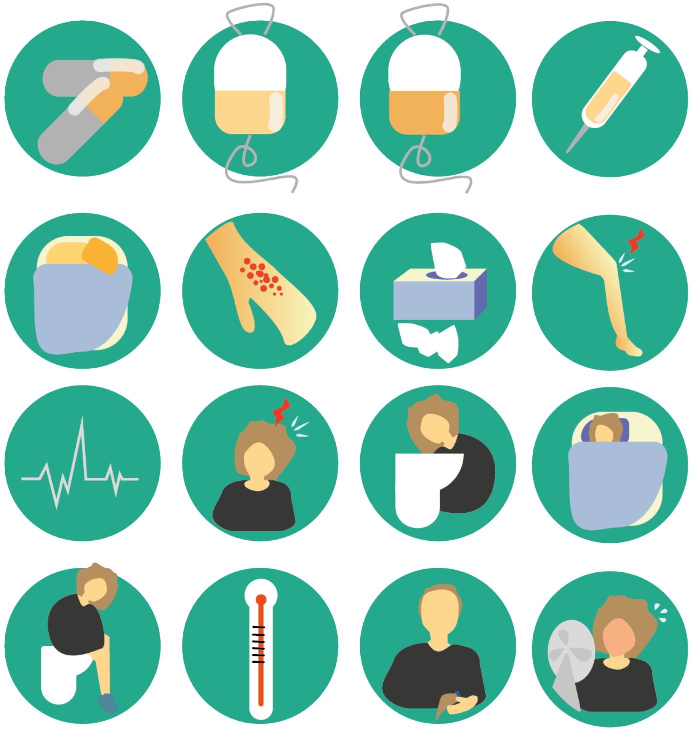

Assignment 2

You see several statements on the next page.

The assignment:

- Choose some statements that are important to you
- Cut these out and stick them in the place that feels right for you in the area on the next page

What is important to you?
Drag the statements (you don't have to use all the statements)

Whether I often have to go to the hospital with this treatment
How long the treatment lasts
What the chance of side-effects is with this treatment
What the chance of late effects is with this treatment
To what extent the treatment increases the chance of living as long as possible
To what extent the treatment affects my desire to have children
To what extent I can just stop this treatment
To what extent the treatment affects my role in my family
To what extent the treatment affects my role in my social network
To what extent the treatment affects my work
How my family feels about the treatment
To what extent the treatment affects my sex drive
How sick I am going to feel from the treatment
To what extent the treatment affects my mental health
To what extent the treatment affects my physical health
To what extent the treatment affects my hobbies

Most important

Least important

Feedback on assignment 1 and 2

How did you feel about expressing what is important to you in these two ways?

Assignment 1: ....

Assignment 2: ....

Assignment 3

Mapping **preferences** through statements can be designed in various ways.
On the next pages, you will see 7 different examples. These are now examples on paper, but it will eventually be an online assignment.

These examples are currently about the format 'way/form' of filling in and not about the content.

Assignment:

- View the 7 different examples (you don't have to complete the examples)
- For each example, fill in positive and negative points about the format way/form of filling in
- If necessary, fill in what else you notice about this example

1

What is important to you?
Drag the statements (you don't have to use all the statements)

- Whether I often have to go to the hospital with this treatment
- How long the treatment lasts
- What the chance of side-effects is with this treatment
- What the chance of late effects is with this treatment
- To what extent the treatment increases the chance of living as long as possible
- To what extent I can just stop this treatment
- To what extent the treatment affects my role in my family
- To what extent the treatment affects my role in my social network
- To what extent the treatment affects my work
- To what extent the treatment affects my hobbies
- How my family feels about the treatment
- How the treatment affects my vitality
- Add yourself ...
- Add yourself ...

Most important

Least important

Positive

Negative

Other

2

What is important to you?

Drag the statements (you don't have to use all the statements)

Whether I often have to go to the hospital with this treatment
How long the treatment lasts
What the chance of side-effects is with this treatment
To what extent the treatment increases the chance of living as long as possible
To what extent the treatment affects my role in my family
To what extent the treatment affects my role in my social network
To what extent the treatment affects my work
How my family feels about the treatment
How the treatment affects my vitality
Add yourself...

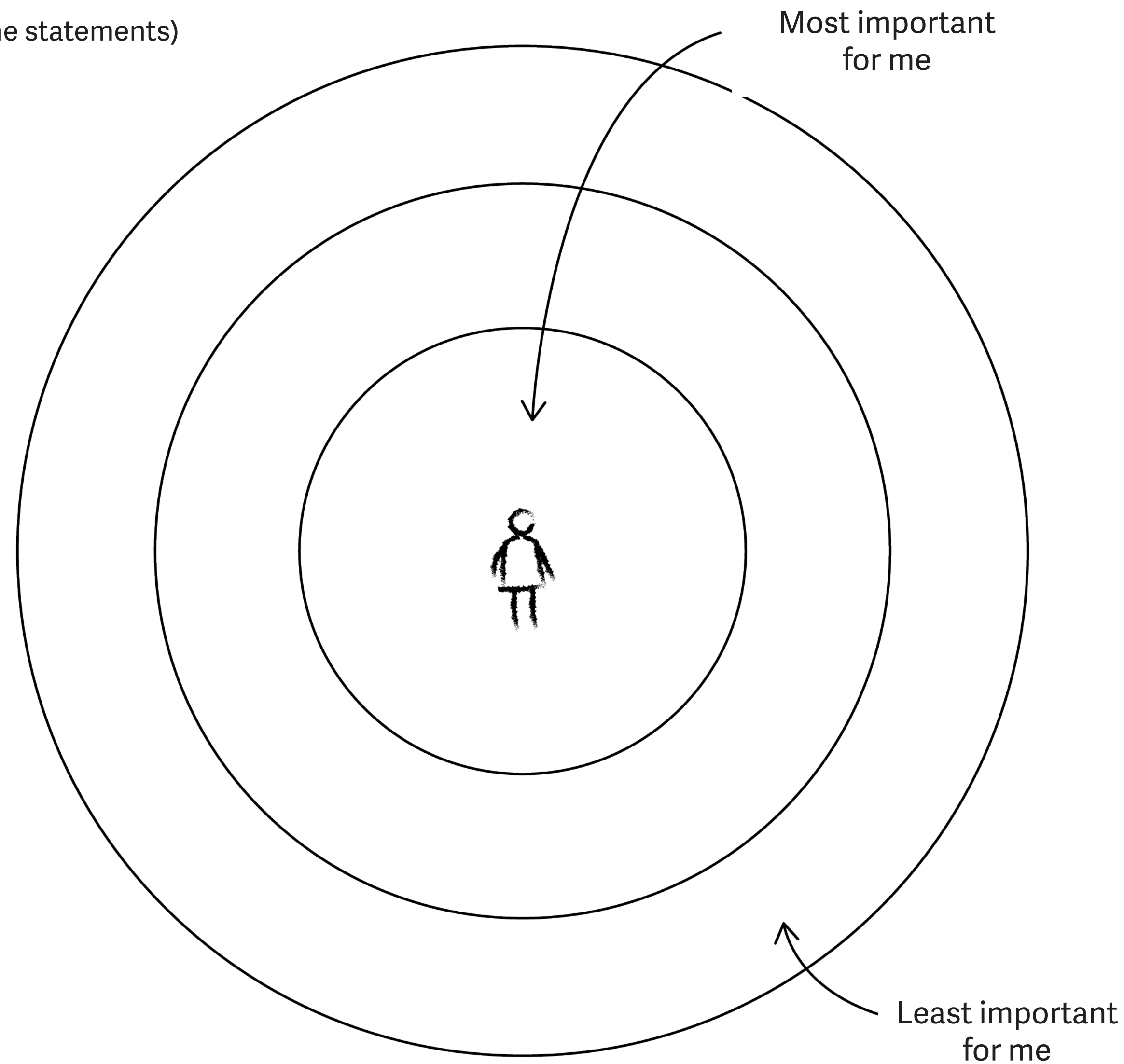

Positive	Negative	Other

3

Open questions:

Please fill in below which side-effects and late effects you are most concerned about.

If your doctor or specialized nurse knows what is important in your life, e.g. your family, your work, or your hobbies, he or she can better work with you in decision-making.

Your doctor or specialized nurse can work better with you if he or she knows what concerns you most or what fears you. You can enter this here:

Your doctor or specialized nurse can work better with you if he or she knows what your loved ones are concerned about. You can enter this here:

Positive	Negative	Other

4

Drag the statements (ranking)

What is important to you?
Drag the statements (1 = most important & 10 = Least important)

1. Whether I often have to go to the hospital with this treatment
2. How long the treatment lasts
3. What the chance of side-effects is with this treatment
4. What the chance of late effects is with this treatment
5. To what extent the treatment increases the chance of living as long as possible
6. To what extent the treatment affects my desire to have children
7. To what extent the treatment affects my mental health
8. To what extent the treatment affects my physical health
9. Add when needed
10. Add when needed

Positive

Negative

Other

5 Pull

What is most important?
You can decrease or increase the bars from unimportant to very important

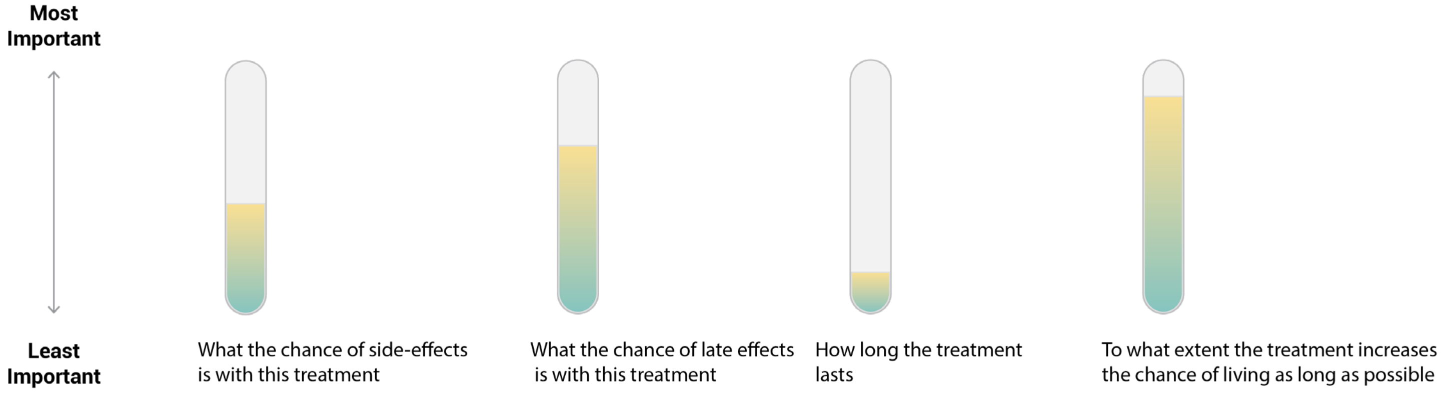

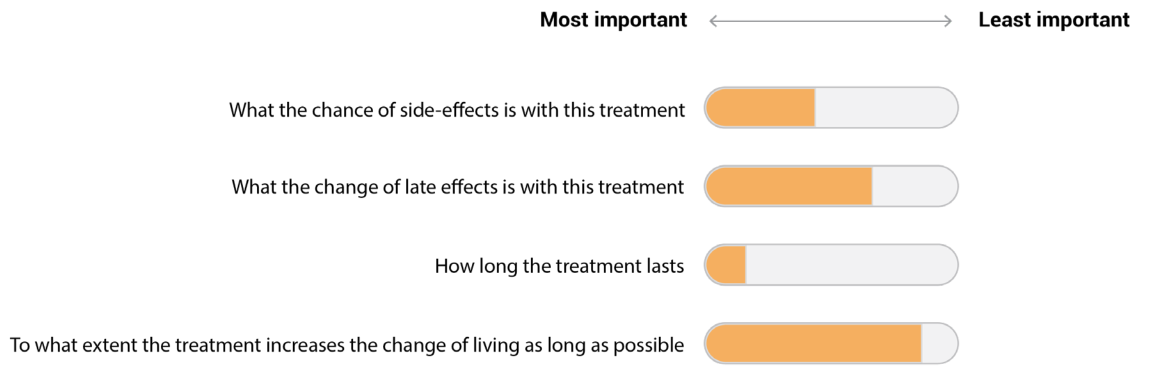

Positive

Negative

Other

6 Move slider

What is most important to you?
You can move the slider to the statement that suits you best.
If you have no opinion, just leave the slider in the middle.

I don't want unpleasant treatments with a risk of side-effects and late effects. Even though I might die sooner. .

I want to live as long as possible. Even though I will suffer from side-effects and perhaps late effects from treatment.

5 4 3 2 1 0 1 2 3 4 5

Agree | No opinion | Agree

Schuifje verplaatsen:

I want to go to the hospital as little as possible.

I don't mind going to the hospital often.

5 4 3 2 1 0 1 2 3 4 5

Agree | No opinion | Agree

Positive

Negative

Neutral

7 Weigh-house

What is more important to you?
You can place the blocks on the scale.
The heavier (more important) a statement is for you, the more blocks you place with this statement. If you don't have an opinion, just leave the scale like this.

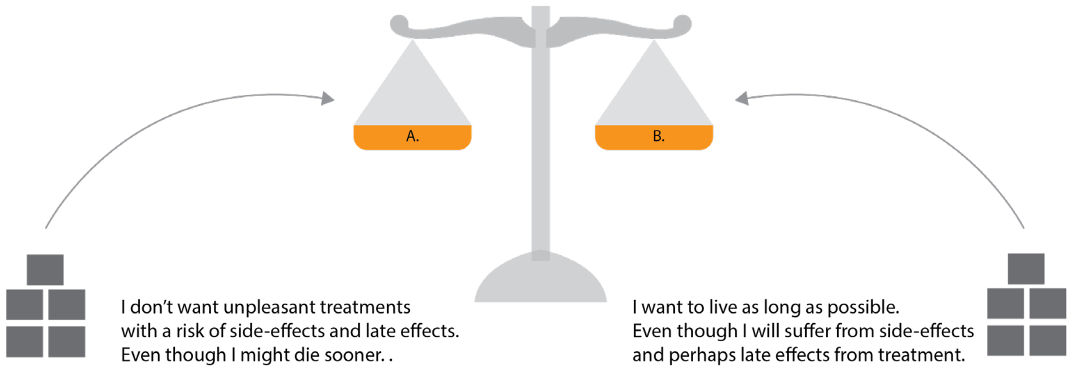

Wat weegt zwaarder voor u?

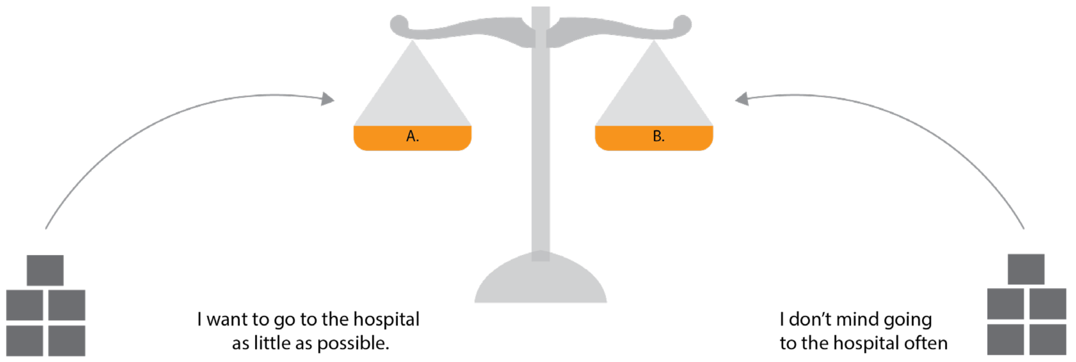

Positive	Negative	Neutral

Feedback on assignment 4

Check out one last time the examples of how to display the preferences.

Which example of an assignment helps you the **most** to reflect your preferences?

Example: …

Because:…

Which example of an assignment helps you the **least** to reflect your preferences?

Example: …

Because:…

Assignment 4

In addition to preferences or values, the chance of **side-effects** and late effects also plays a role in deciding about undergoing additional treatment.

In the previous meeting, it emerged that it is important to know how often these side-effects and late effects occur.

On the following pages, you will see 6 examples of different ways in which the chance of side-effects or late effects can be displayed.

Assignment:

- View the examples on how to display the chances of side-effects and late effects.
- For each example, fill in positive and negative points about the way of displaying the chances.
- If necessary, fill in what else you notice about this example

1

How common is this side-effect?

Nausea

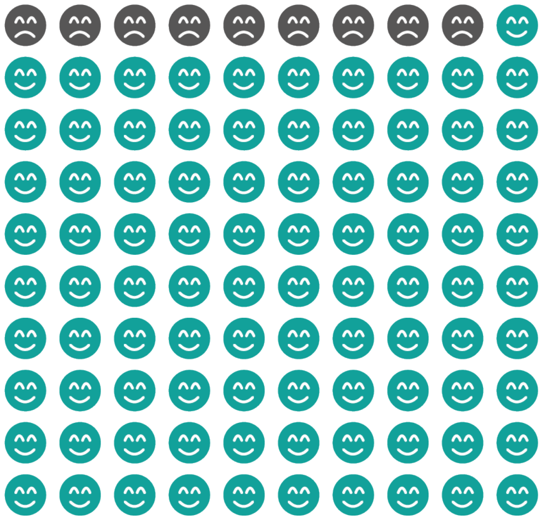

9 out of 100 women
have this side effect

Fatigue

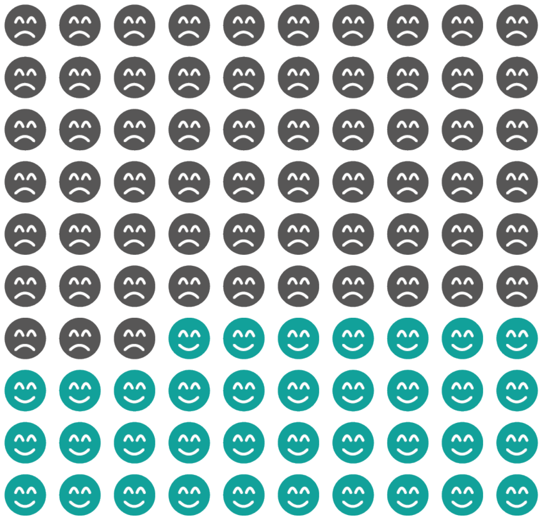

63 out of 100 women
have this side effect

Positive:

Negative:

Other:

2

How common is this side-effect?

Nausea

Rarely	Sometimes	**Often**	Very often

↑

9 out of 100 women
have this side effect

Fatigue

Rarely	Sometimes	Often	**Very often**

↑

63 out of 100 women
have this side effect

Very often: 10 or more in 100 women
Often: 1 to 10 in 100 women
Sometimes: 1 to 10 in 1000 women
Rarely: 1 to 10 to 10.000 woman

Positive::

Negative:

Other:

3

How common is this side-effect?

Nausea

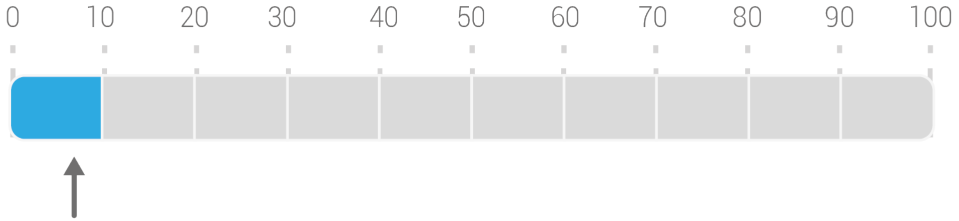

9 out of 100 women
have this side effect

Fatigue

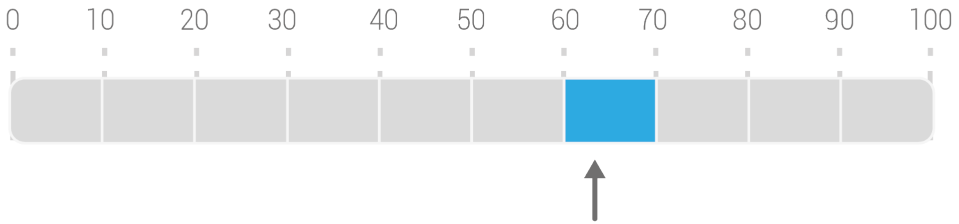

63 out of 100 women
have this side effect

Positive::

Negative:

Other:

4

How common is this side-effect?

Nausea

Rarely | Sometimes | **Often** | Very often

9 out of 100 women
have this side effect

Fatigue

Rarely | Sometimes | Often | **Very often**

63 out of 100 women
have this side effect

Very often: 10 or more in 100 women
Often: 1 to 10 in 100 women
Sometimes: 1 to 10 in 1000 women
Rarely: 1 to 10 to 10.000 woman

Positive::

Negative:

Other:

5

How common is this side-effect?

Nausea

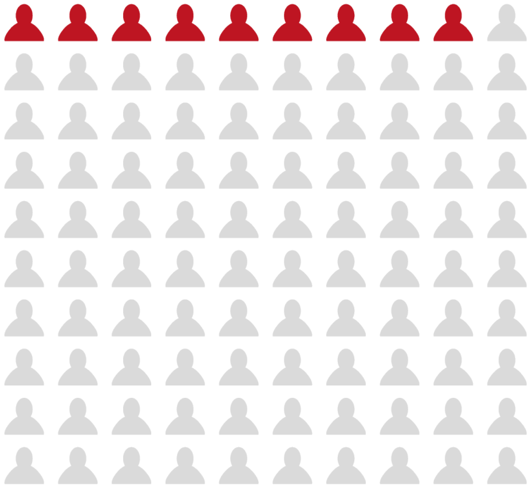

9 out of 100 women
have this side effect

Fatigue

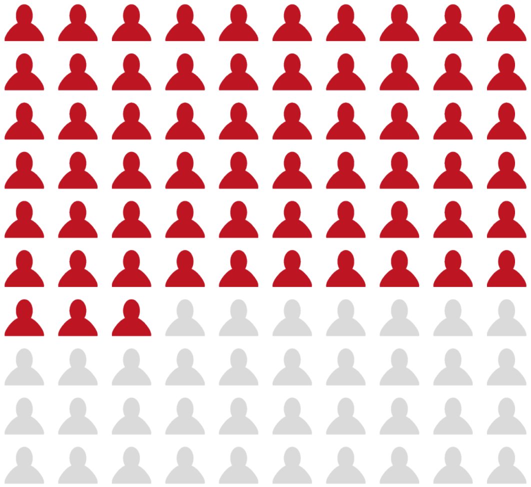

63 out of 100 women
have this side effect

Positive::

Negative:

Other:

6

How common is this side-effect?

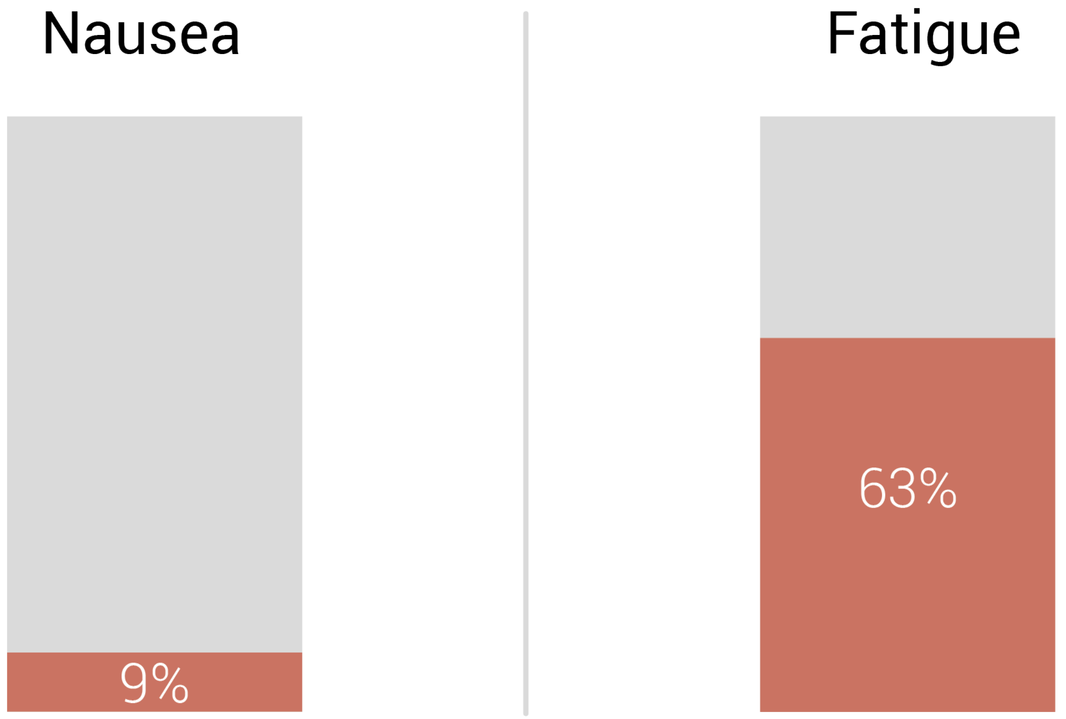

9% of the women
has this side-effect

63% of the women
has this side-effect

Positive::

Negative:

Other:

Feedback on assignment 4

Check out one last time the examples of how to display the chances.

Which example would help you **most** if you would want to know the chance of a side-effect?

Example: …

Because:…

Which example of an assignment would help you **least** if you would want to know the chance of a side-effect?

Example: …

Because:…

Questions related to assignment 4

Finally, we would like to ask you a few more questions about the examples.

Example 1

How **likely** do you think you are to get the side-effect of fatigue?

Not likely at all						Very likely
1	2	3	4	5	6	7

How **concerned** are you about getting the fatigue side-effect?

Not concerned at all						Very concerned
1	2	3	4	5	6	7

Fatigue

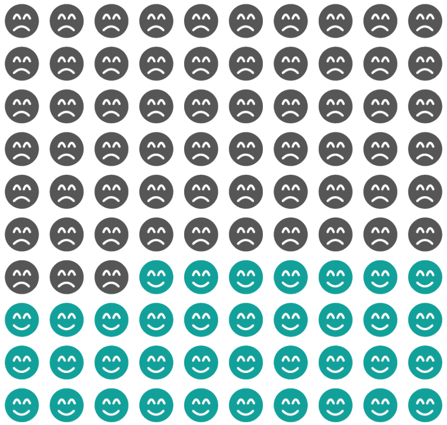

63 out of 100 women have this side effect

Example 2

How **likely** do you think you are to get the side-effect of fatigue?

Not likely at all — Very likely

1 2 3 4 5 6 7

How **concerned** are you about getting the fatigue side-effect?

Not concerned at all — Very concerned

1 2 3 4 5 6 7

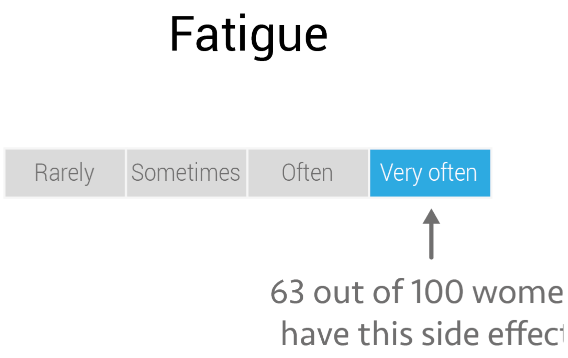

Example 3

How **likely** do you think you are to get the side-effect of fatigue?

Not likely at all — Very likely

1 2 3 4 5 6 7

How **concerned** are you about getting the fatigue side-effect?

Not concerned at all — Very concerned

1 2 3 4 5 6 7

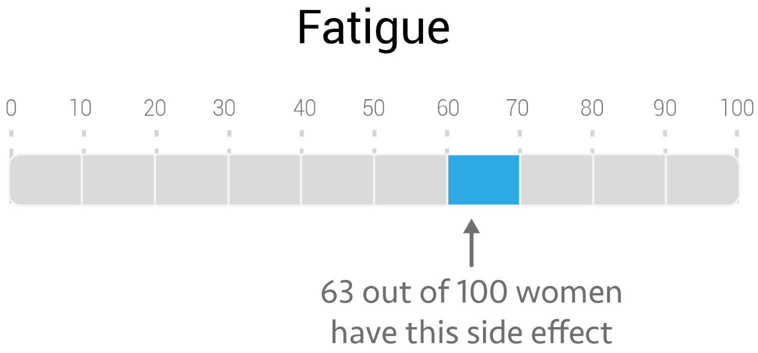

Example 4

How **likely** do you think you are to get the side-effect of fatigue?

Not likely at all — Very likely

1 2 3 4 5 6 7

How **concerned** are you about getting the fatigue side-effect?

Not concerned at all — Very concerned

1 2 3 4 5 6 7

Fatigue

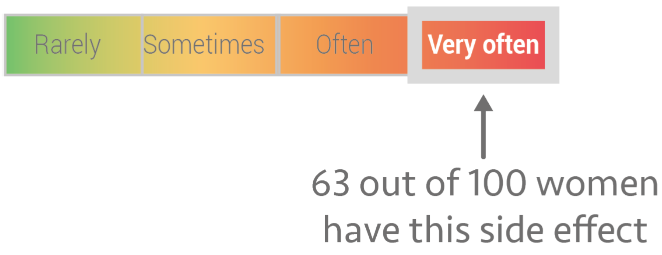

Example 5

How **likely** do you think you are to get the side-effect of fatigue?

Not likely at all — Very likely

1 2 3 4 5 6 7

How **concerned** are you about getting the fatigue side-effect?

Not concerned at all — Very concerned

1 2 3 4 5 6 7

Fatigue

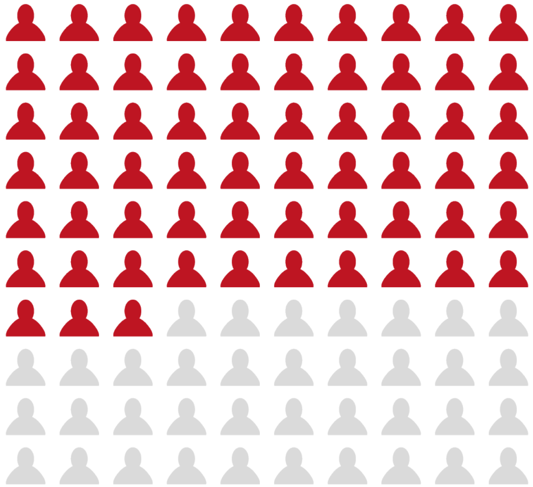

63 out of 100 women
have this side effect

Example 6

How **likely** do you think you are to get the side-effect of fatigue?

Not likely at all — Very likely

1 2 3 4 5 6 7

How **concerned** are you about getting the fatigue side-effect?

Not concerned at all — Very concerned

1 2 3 4 5 6 7

Fatigue

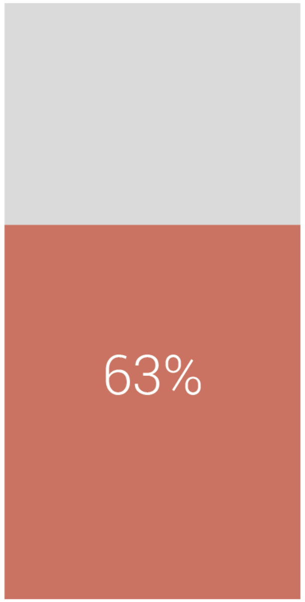

63% of the women has this side-effect

Thank you!

Thanks for completing!

Do you want to share photos of the assignments via e-mail?

If this does not work, you can return the assignments to us with the return envelope.
